# Clinically relevant morphological structures in breast cancer represent transcriptionally distinct tumor cell populations with varied degrees of epithelial-mesenchymal transition and CD44^+^CD24^-^ stemness

**DOI:** 10.18632/oncotarget.18022

**Published:** 2017-05-19

**Authors:** Evgeny V. Denisov, Nikolay A. Skryabin, Tatiana S. Gerashchenko, Lubov A. Tashireva, Jochen Wilhelm, Mikhail A. Buldakov, Aleksei A. Sleptcov, Igor N. Lebedev, Sergey V. Vtorushin, Marina V. Zavyalova, Nadezhda V. Cherdyntseva, Vladimir M. Perelmuter

**Affiliations:** ^1^ Laboratory of Molecular Oncology and Immunology, Cancer Research Institute, Tomsk National Research Medical Center, Russian Academy of Sciences, 634050, Tomsk, Russian Federation; ^2^ Laboratory for Translational Cellular and Molecular Biomedicine, Tomsk State University, 634050, Tomsk, Russian Federation; ^3^ Department of Organic Chemistry, Tomsk State University, 634050, Tomsk, Russian Federation; ^4^ Laboratory of Cytogenetics, Research Institute of Medical Genetics, Tomsk National Research Medical Center, Russian Academy of Sciences, 634050, Tomsk, Russian Federation; ^5^ Laboratory of Human Ontogenetics, Tomsk State University, 634050, Tomsk, Russian Federation; ^6^ Department of General and Molecular Pathology, Cancer Research Institute, Tomsk National Research Medical Center, Russian Academy of Sciences, 634050, Tomsk, Russian Federation; ^7^ Department of Internal Medicine, German Center for Lung Research, Excellence Cluster Cardio-Pulmonary System, Universities of Giessen and Marburg Lung Center, D-35392, Giessen, Germany; ^8^ Laboratory of Population Genetics, Research Institute of Medical Genetics, Tomsk National Research Medical Center, Russian Academy of Sciences, 634050, Tomsk, Russian Federation; ^9^ Department of Pathological Anatomy, Siberian State Medical University, 634050, Tomsk, Russian Federation

**Keywords:** breast cancer, tumor heterogeneity, epithelial-mesenchymal transition, cancer invasion, cancer stem cell

## Abstract

Intratumor morphological heterogeneity in breast cancer is represented by different morphological structures (tubular, alveolar, solid, trabecular, and discrete) and contributes to poor prognosis; however, the mechanisms involved remain unclear. In this study, we performed 3D imaging, laser microdissection-assisted array comparative genomic hybridization and gene expression microarray analysis of different morphological structures and examined their association with the standard immunohistochemistry scorings and CD44^+^CD24^-^ cancer stem cells. We found that the intratumor morphological heterogeneity is not associated with chromosomal aberrations. By contrast, morphological structures were characterized by specific gene expression profiles and signaling pathways and significantly differed in progesterone receptor and Ki-67 expression. Most importantly, we observed significant differences between structures in the number of expressed genes of the epithelial and mesenchymal phenotypes and the association with cancer invasion pathways. Tubular (tube-shaped) and alveolar (spheroid-shaped) structures were transcriptionally similar and demonstrated co-expression of epithelial and mesenchymal markers. Solid (large shapeless) structures retained epithelial features but demonstrated an increase in mesenchymal traits and collective cell migration hallmarks. Mesenchymal genes and cancer invasion pathways, as well as Ki-67 expression, were enriched in trabecular (one/two rows of tumor cells) and discrete groups (single cells and/or arrangements of 2-5 cells). Surprisingly, the number of CD44^+^CD24^-^ cells was found to be the lowest in discrete groups and the highest in alveolar and solid structures. Overall, our findings indicate the association of intratumor morphological heterogeneity in breast cancer with the epithelial-mesenchymal transition and CD44^+^CD24^-^ stemness and the appeal of this heterogeneity as a model for the study of cancer invasion.

## INTRODUCTION

Intratumor morphological heterogeneity is a common phenomenon for many cancers and is represented by various morphological structures (named also as histological, invasive, and infiltrative patterns) that reflect the different architectural arrangements of tumor cells. In dependence on the prevalence of a certain type of morphological structure, cancers are classified to distinct histological forms with a specific prognosis and response to therapy. However, different histological patterns can coexist within the same tumors [1, 2]. Moreover, certain morphological structures are taken into account in cancer grading and are closely related to therapy response and disease prognosis [3–6].

**Figure 1:**
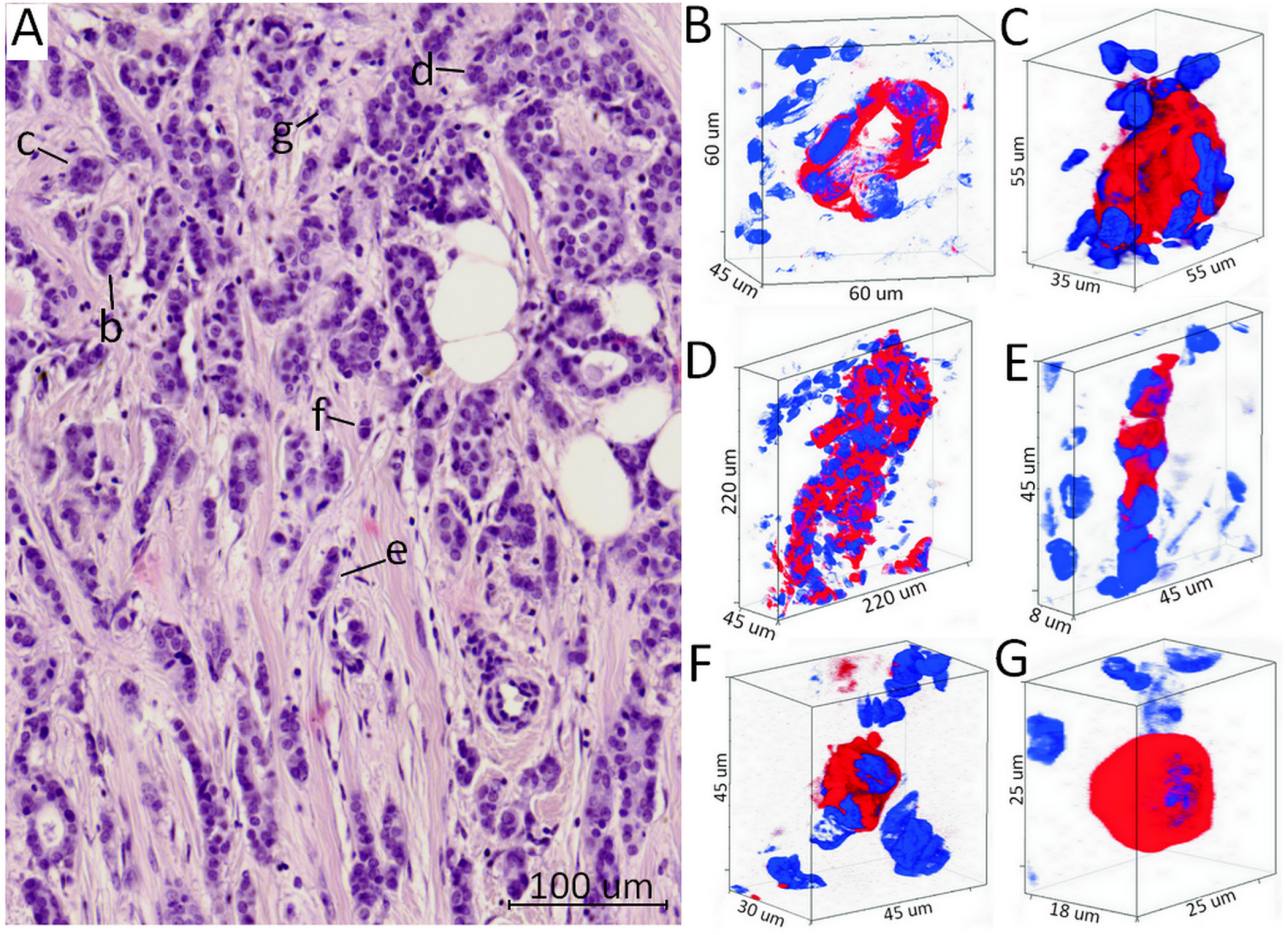
Different morphological structures of IC NST **(A)** Hematoxylin and eosin stained section with different morphological structures: tubular **(b)**, alveolar **(c)**, solid **(d)**, trabecular **(e)**, and discrete **(f, g)**. **(B-G)** Three-dimensional immunofluorescence images of different morphological structures. Tubular structures **(B)** are represented by the tube-shaped aggregations of tumor cells. Alveolar structures **(C)** have a rounded (spheroidal) shape and contain up to 30 tumor cells. Solid structures **(D)** are represented by the large shapeless groups of tens and hundreds of tumor cells. Trabecular structures **(E)** are formed by one or two rows of tumor cells. Discrete groups of tumor cells **(F, G)** are defined by arrangements of 2-5 cells and/or single cells. Red color indicates the cytoplasmic expression of cytokeratin 7 (anti-CK7 antibodies, clone OV-TL, Dako, Denmark), blue – DAPI staining (nucleus).

Breast cancer (BC) is no exception; there is striking morphological diversity within breast tumors. The most common form of BC – invasive carcinoma of no special type (IC NST) – presents an extremely diverse invasive component, within which tumor cells may be single or arranged in either small groups or multicellular structures: tubular (hollow-like), alveolar (morula-like), solid, and trabecular structures (Figure 1A) [7, 8]. The number of different types of morphological structures in breast tumors varies from case to case and depends on the molecular subtype of BC [9]. Different morphological structures were found to differ in the expression of cell adhesion and drug resistance genes [10, 11] and the distribution of tumor-associated macrophages and fibroblasts in their microenvironment [12]. The intratumor morphological heterogeneity of BC appeared to be involved in cancer metastasis and chemotherapy efficiency. The presence of alveolar structures in breast tumors was associated with a high frequency of lymph node involvement [8, 9]. Patients either with alveolar/trabecular structures or with all types of morphological structures demonstrated a poor response to neoadjuvant chemotherapy [11, 13]. Moreover, alveolar and trabecular structures were associated with poor metastasis-free survival in BC patients [14].

At present, there is very limited information on the nature and the mechanisms of intratumor morphological heterogeneity in human cancers, including BC. Moreover, the molecular or cellular factors involved in the role of morphological diversity in cancer progression and therapy efficacy are unknown. It is reasonable to presume that the morphological diversity within breast tumors reflects genetic events. However, the data available are either ambiguous or incomplete because the results have been derived from studies with different histological types of BC as well as various morphological structures [15–17]. Epithelial-mesenchymal transition (EMT) and cancer stem cells (CSCs) may be involved in the heterogeneity of the invasive component of breast carcinomas. The repression of epithelial differentiation and the acquisition of stemness appear to be intimately interconnected processes [18, 19]. EMT results in the generation of cells with epithelial-mesenchymal (hybrid) and mesenchymal phenotypes [20]. Due to changes in cell adhesion occurring within EMT, these cell states may look morphologically like different architectural arrangements of tumor cells.

Thus, in this study, we hypothesized that the intratumor morphological heterogeneity in BC may be related to genetic and gene expression changes as well as EMT/stemness. To verify this hypothesis, we analyzed chromosomal aberrations, transcriptome changes, and the presence of CD44^+^CD24^-^ CSCs in different morphological structures: tubular, solid, alveolar, and trabecular structures, as well as discrete groups of tumor cells. In addition, we analyzed the three-dimensional (3D) organization of different morphological structures and their association with the standard immunohistochemistry (IHC) scorings in BC – expression of estrogen (ER), progesterone (PR) receptors, HER2, and Ki-67.

## RESULTS

### 3D organization of different morphological structures within breast tumors

Herein, we studied the spatial shape of different morphological structures within breast tumors (Figure 1). Tubular structures were represented as tube-shaped cell aggregations consisting of one row of tumor cells. Some part of these structures demonstrated the irregular shape that was manifested in the formation of more than one row of tumor cells on any side of the structure (Figure 1B, Supplementary Movie 1). Alveolar structures had a rounded (spheroidal) shape and contained up to 30 tumor cells (Figure 1C, Supplementary Movie 2). Solid structures represented groups with different sizes and shapes consisting of tens and hundreds of tumor cells. Some solid structures demonstrated row- or sheet-like protrusions of tumor cells (Figure 1D, Supplementary Movie 3). Trabecular structures were formed by one or two rows of tumor cells (Figure 1E, Supplementary Movie 4). Discrete groups of tumor cells included both single cells and arrangements of 2-5 cells with distinct epithelial morphology (shape) (Figure 1F, 1G, Supplementary Movie 5-6). All types of structures were both surrounded by a sufficient number of cells in the tumor microenvironment and located separately from immune and stromal cells.

Thus, we showed that different morphological structures are characterized by specific 3D shapes and varied number of tumor cells that compose them.

### The hormonal receptor, HER2 and Ki-67 expression status of different morphological structures in breast tumors

In this section, we analyzed differences in ER, PR, HER2, and Ki-67 expression between different morphological structures in breast tumors (Supplementary Tables 1 and 2). No statistically significant differences in ER and HER2 expression were found. However, discrete groups showed a tendency to decrease in ER expression compared to other structures (p=0.05; Supplementary Table 1). PR expression was the lowest in discrete groups (p<0.05 vs. alveolar/tubular structures). The lowest proliferative activity as identified by Ki-67 expression was found in tubular structures (0.1<p<0.05 vs. solid, trabecular, and discrete groups; Supplementary Table 1). In contrast, the highest Ki-67 activity was shown in trabecular and discrete groups; however, the differences were or tended to be statistically significant only in comparison with tubular structures (Supplementary Table 1). Interestingly, an increase in Ki-67 in tubular and alveolar structures was associated with a high frequency of lymph node involvement in patients with luminal A breast cancer (p<0.05; data not shown).

Thus, we found that different morphological structures do not differ from each other in terms of ER and HER2 status, but demonstrate significant differences in PR and Ki-67 expression.

### The association of different morphological structures of breast tumors with chromosomal aberrations

In this part of the study, we investigated the involvement of chromosomal aberrations in the intratumor morphological heterogeneity of BC. In particular, we analyzed chromosomal abnormalities in different morphological structures of three regions of each breast tumor (n=3).

**Figure 2:**
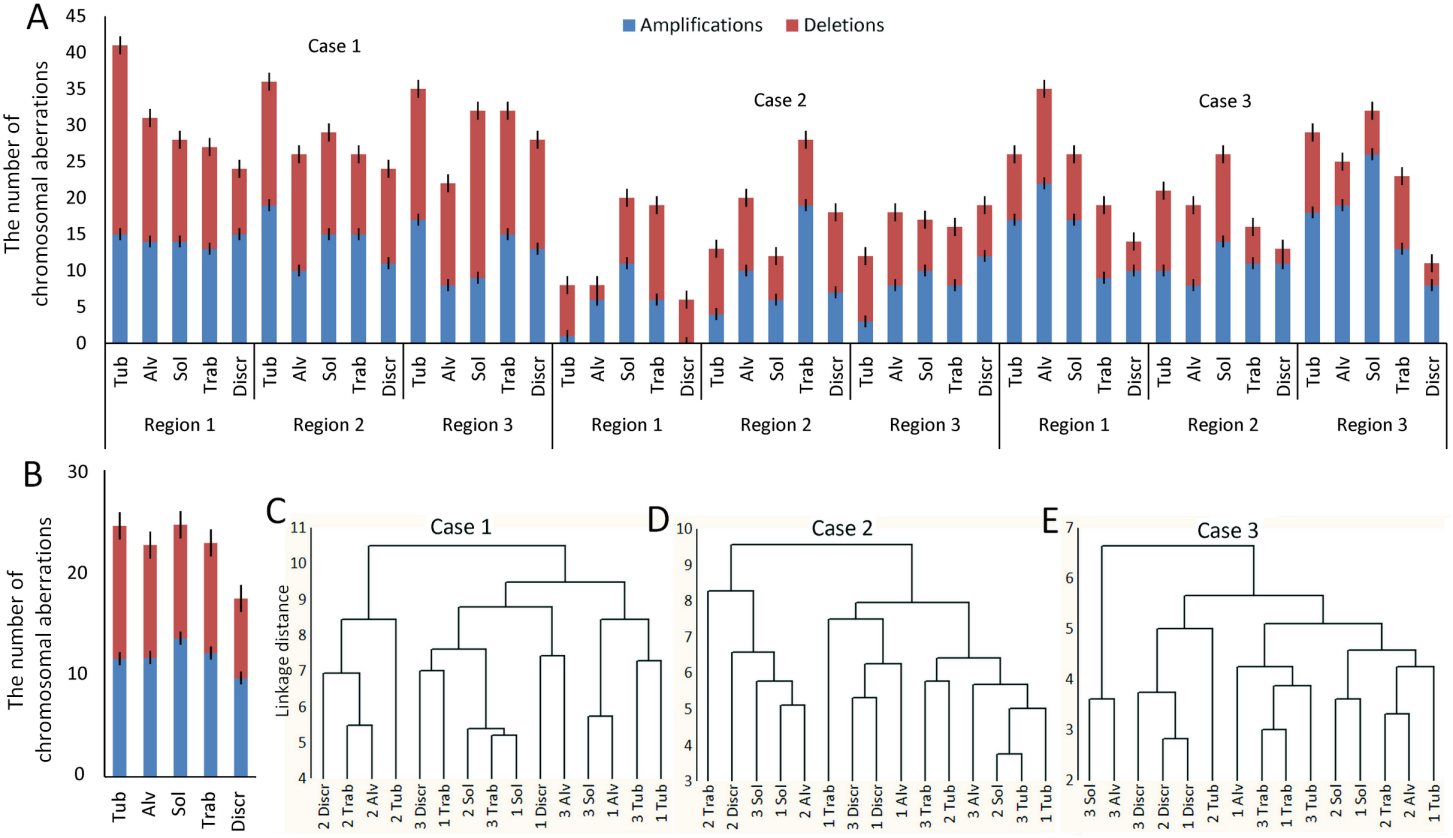
The association between different morphological structures of IC NST and chromosomal aberrations **(A)** The number of chromosomal aberrations in the different morphological structures of three regions (1-3) of each breast tumor (cases #1-3). **(B)** The mean number of chromosomal aberrations in the different morphological structures. **(C-E)** Cluster analysis of the measurement of similarity of different structures of three regions (1-3) of each breast tumor (cases #1-3) with each other with regard to the number of common chromosomal aberrations. The figure was prepared based on the data of aCGH analysis of different morphological structures of three BC cases (#1-3 in Supplementary Table 7). Tub, tubular; Alv, alveolar; Sol, solid; Trab, trabecular; Discr, discrete.

The number of unbalanced chromosomal aberrations in the different structures from various regions ranged from 6 to 41 (mean ± standard deviation, 22.4±8.2; Figure 2A). The lowest number of abnormalities was found in discrete groups of tumor cells, and the most in solid and tubular structures (Figure 2B).

Common chromosomal rearrangements for all of the analyzed structures (n=15) from three tumor regions were observed only in cases 1 and 3 (Table 1). In the first patient, there was the gain of chromosome region 11q13.5-q14.1, in the third – the gain of 1q and monosomy of chromosome 13. In the second patient, one aberration, monosomy of chromosome 6, was found in 80% (12/15) of the samples. In cases 1 and 3, the presence of multiple common chromosomal abnormalities suggests their early manifestation in tumorigenesis, whereas the absence of such frequent aberrations in the second patient may indicate the delayed development of chromosomal instability. Interestingly, the gain of 22q11.2, which was one of the most frequent chromosomal aberrations in case 3 (93.3% of samples), was also detected in cases 1 (66%) and 2 (20%). This amplification affects only one gene, which encodes miR-650. Previous studies described the involvement of miR-650 expression in the poor prognosis of various cancers [21, 22]. In BC, the miR-650 amplification has been recently suggested to be involved in triggering EMT [23].

**Table 1:** Common chromosomal aberrations for morphological structures isolated from three regions of each breast tumor (n=3)

Case	Chromosomal aberrations and their frequency			
	100.0%	93.3%	86.6%	80.0%
1	amp 11q13.5-q14.1	amp 5q35.1-q35.3 del 7q22.1-q32.1 amp 17q22-q25.3 monosomy 22	del 2q31.1-q37.3 del 17p11.2-p13.3	del 5q33.2-q35.1
2	-	-	-	monosomy 6
3	amp 1q monosomy 13	del 1p13.1-p31.1 amp 22q11.22	amp 1p12 del 17p	amp 17q

amp: amplification; del: deletion.

To analyze the similarity of the morphological structures in the spectrum of chromosomal abnormalities, we conducted a cluster analysis (Figure 2C-2E). No significant association between the morphological structures and chromosomal abnormalities were found. Nevertheless, in the first patient, most of the morphological structures (except the solid structures) in the second tumor region were clustered together (Figure 2C). This fact indicates a common ancestor of these structures and a tumor origin that is independent from two other regions of the tumor. The most noteworthy results were found in the case 3 (Figure 2E). Discrete groups of tumor cells from three tumor regions were clustered together, which indicated the presence of common chromosomal aberrations. In particular, all of the discrete groups of tumor cells in case 3 had a duplication of 11p13 region, which contains only one gene – *PAX6*. This gene has been found to promote BC cell proliferation and tumor progression [24].

Overall, these results suggest the intratumor morphological heterogeneity in BC is not associated with chromosomal abnormalities.

### Cluster analysis of transcript expression profiles of different morphological structures in breast tumors

Herein we questioned whether different morphological structures differ in their transcript expression profile from each other and if there is a correlation between the same structures obtained from different breast tumors.

**Figure 3:**
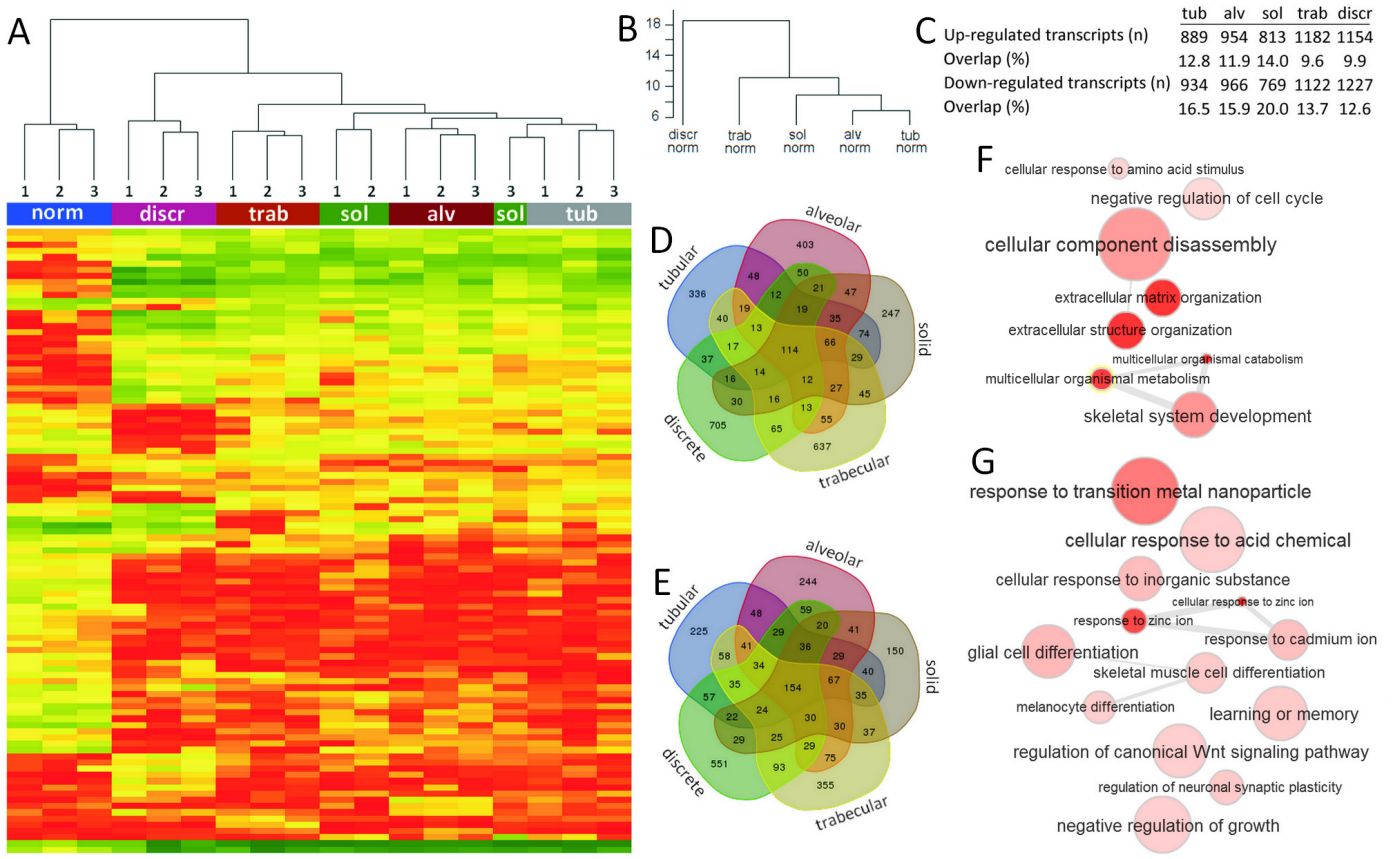
Expression profile of different morphological structures of IC NST **(A)** Heat map and unsupervised hierarchical clustering analysis of the 100 most significantly dysregulated transcripts in morphological structures compared to normal breast epithelia. **(B)** Unsupervised hierarchical clustering analysis of the whole transcriptome expression in morphological structures normalized to normal breast epithelia. The y-axis shows the height (inter-cluster distance) between these different clusters. **(C)** The number of up- and down-regulated transcripts and the percentage of overlapping transcripts in each morphological structure. **(D, E)** Venn diagrams of up- and down-regulated transcripts in the morphological structures. **(F, G)** GO enrichment analysis of up- and down-regulated overlapping transcripts of the morphological structures. The figure was prepared based on the data of gene expression microarray profiling of different morphological structures of three BC cases (#1-3 in Supplementary Table 7). Tub: tubular; Alv: alveolar; Sol: solid; Trab: trabecular; Discr: discrete; Norm: normal breast epithelia; 1-3: case numbers.

Cluster analysis of the top 100 transcripts with the lowest p values (FDR) showed the similarity of morphological structures of the same type obtained from three different breast tumors. The exception was the solid structures from case 3, which was clustered with tubular structures. The similarity was observed for all samples of histologically normal ducts isolated from tumor-adjacent breast tissue of three cases. Interestingly, the tubular structures were more similar with the alveolar structures than with other structures. Discrete groups of tumor cells significantly differed in their transcript expression profile from other structures (Figure 3A). The comparison of transcript expression profiles of the morphological structures normalized to normal breast epithelia confirmed the same continuity. In particular, the differences in transcript expression were increased in ascending order: tubular = alveolar, solid, trabecular, and discrete groups (Figure 3B).

Note, different structures obtained from each of three breast tumors and undergoing gene expression profiling demonstrated similar ER expression and surprisingly did not differ in PR status as compared to the aforementioned findings indicated in Supplementary Table 1. Nevertheless, Ki-67 expression also significantly varied between different structures reaching maximum values in discrete groups (data not shown).

Taken together, these findings indicate that different morphological structures represent transcriptionally distinct tumor cell populations.

### Common and specific transcripts in different morphological structures of breast tumors

In this part of the study, we identified and analyzed transcripts expressed both in all of the morphological structures and specifically in certain types of structures. Gene expression data generated by microarrays were validated using quantitative reverse transcription PCR (qRT-PCR). The transcript levels obtained by the microarrays and qRT-PCR were positively correlated in a linear regression model (r^2^ = 0.838; *p*<0.05; Supplementary Figure 1).

Different morphological structures showed a distinct number of up- and down-regulated transcripts in comparison with normal breast epithelia. The list of genes whose expression was at an p<0.05 (FDR) is shown in Supplementary Table 3. Many up-regulated genes (p<0.05, FDR) in the morphological structures were related to the regulation of extracellular matrix (ECM) organization mainly via the activation of collagen (*COL1A1, COL1A2, COL3A1* etc.) and fibronectin (*FN1* and *THC2507047*) transcripts. In addition, almost all of the structures showed overexpression of the *S100A7*, *SFRP4*, and *HMGB3* genes that are involved in the regulation of cell cycle, growth, progression, differentiation, and maintaining cell stemness. The down-regulated transcripts included uncharacterized LOC102723505 (RNA gene affiliated with the ncRNA class), Chromosome 2 Open Reading Frame 40 (*C2orf40*), which was previously characterized as a tumor suppressor in BC [25], the *KIT* gene involved in the regulation of a number of cellular processes (cell survival and proliferation, stem cell maintenance etc.) and some others.

Table 2 lists the transcripts differentially expressed (p<0.05, FDR) between the different morphological structures (the expression values were not normalized to normal breast epithelia). In particular, the trabecular structures demonstrated significant overexpression of only *PKDREJ*, which encodes the polycystin (PKD) family receptor for egg jelly. Discrete groups of tumor cells showed a set of different up- and down-regulated genes. For example, we found overexpression of *MAMDC2* and *ADAMTS12* as well as underexpression of *KIF1B* and *GPR160*. Interestingly, discrete groups of tumor cells were also characterized by extremely high expression of *CD248* (endosialin), which was previously described to be expressed in cells of mesenchymal origin (e.g., vascular smooth muscle cells, myofibroblasts, etc.) and by tumor cells themselves [26].

**Table 2:** Genes up- and down-regulated only in trabecular structures and discrete groups of tumor cells

Gene symbol	Full gene name	Location	Log-fold change	Adjusted P-value
Trabecular structures				
Upregulated genes				
*PKDREJ*	polycystin (PKD) family receptor for egg jelly	22q13.31	4.471	0.002
Discrete groups of tumor cells				
Upregulated genes				
*MAMDC2*	MAM domain containing 2	9q21.2	5.439	0.016
*ADAMTS12*	ADAM metallopeptidase with thrombospondin type 1 motif 12	5q35	4.982	0.012
*CD248*	CD248 molecule (endosialin)	11q13	4.378	0.016
*C1QTNF3*	C1q and tumor necrosis factor related protein 3	5p13	4.267	0.012
*CRCT1*	cysteine rich C-terminal 1	1q21	4.191	0.012
*EMCN*	endomucin	4q22.1	3.978	0.016
*APOBR*	apolipoprotein B receptor	16p11.2	3.768	0.040
*MMP13*	matrix metallopeptidase 13	11q22.3	3.548	0.031
*GPR4*	G protein-coupled receptor 4	19q13.3	3.335	0.034
*CYP46A1*	cytochrome P450 family 46 subfamily A member 1	14q32.2	3.049	0.019
*SCARNA1*	small Cajal body-specific RNA 1	1p35.3	2.824	0.034
*CEACAM21*	carcinoembryonic antigen related cell adhesion molecule 21	19q13.2	2.470	0.043
*TIMP2*	TIMP metallopeptidase inhibitor 2	17q25	2.112	0.043
Downregulated genes				
*CCDC117*	coiled-coil domain containing 117	22q12.1	-2.707	0.032
*DAZAP1*	DAZ associated protein 1	19p13.3	-3.211	0.033
*SLC12A2*	solute carrier family 12, member 2	5q23.3	-3.273	0.016
*RNASEH2B*	ribonuclease H2 subunit B	13q14.3	-3.844	0.019
*FUT8*	fucosyltransferase 8 (alpha (1,6) fucosyltransferase)	14q24.3	-3.881	0.022
*SVIP*	small VCP/p97-interacting protein	11p14.3	-3.976	0.039
*SMIM7*	small integral membrane protein 7	19p13.11	-3.999	0.043
*HINT3*	histidine triad nucleotide binding protein 3	6q22.33	-4.026	0.039
*FGD6*	FYVE, RhoGEF and PH domain containing 6	12q23.1	-4.109	0.022
*IL20RA*	interleukin 20 receptor subunit alpha	6q23.3	-4.218	0.023
*RALGAPA1*	Ral GTPase activating protein catalytic alpha subunit 1	14q13.2	-4.326	0.016
*TIPARP*	TCDD-inducible poly(ADP-ribose) polymerase	3q25.31	-4.349	0.016
*MRPL10*	mitochondrial ribosomal protein L10	17q21.3	-4.388	0.016
*PACS2*	phosphofurin acidic cluster sorting protein 2	14q32	-4.530	0.012
*EHF*	ets homologous factor	11p13	-4.605	0.016
*MTURN*	maturin	7p15.1	-4.649	0.012
*GFM2*	G elongation factor, mitochondrial 2	5q13	-4.822	0.034
*GPR160*	G protein-coupled receptor 160	3q26.2-q27	-5.036	0.019
*KIF1B*	kinesin family member 1B	1p36.22	-5.427	0.016

Thus, we showed that different morphological structures are characterized by expression of both common transcripts associated mainly with the organization of ECM and the regulation of cell growth and specific genes.

### Top-enriched pathways in different morphological structures of breast tumors

Here, we performed pathway analysis of transcripts that were differentially expressed in the different morphological structures of breast tumors. The analysis involved transcript expression profiles of the structures normalized to normal breast epithelia.

We identified up- (n=114; 9.6-14.0%) and down-regulated (n=154; 12.6-20.0%) transcripts that overlapped between the different structures (Figure 3C-3E). The GO enrichment analysis showed that common up-regulated transcripts were mainly involved in the regulation of ECM and cellular metabolism (Figure 3F; Supplementary Table 4). In contrast, the overlapping down-regulated transcripts were predominantly related to the response to metal ions and cell differentiation (Figure 3G; Supplementary Table 5).

Morphological structures were characterized by the regulation of common pathways; however, the statistical significance (p value), the ratio (the percentage of genes involved in the canonical pathway), and other parameters of the association differed among them (Table 3). Tubular structures were associated with the regulation of pathways (acute myeloid leukemia signaling, molecular mechanisms of cancer, etc.) that were more significantly enriched in other structures. Alveolar structures showed more considerable association with the EMT pathway and ErbB and actin cytoskeleton signaling. Solid variants were related mainly to the regulation of the molecular mechanisms of cancer, G2/M DNA damage checkpoint, and PAK signaling, although the DNA damage checkpoint was represented only by up-regulated genes that suggest its activation, and the PAK signaling pathway contained three times more down-regulated than up-regulated genes. Trabecular structures were predominantly characterized by acute phase response, Fc epsilon RI, and PDGF signaling, the latter two of which had significantly more underexpressed than overexpressed genes. Discrete groups of tumor cells demonstrated a significant association with hepatic fibrosis/hepatic stellate cell activation and acute myeloid leukemia signaling, with up-regulated genes predominant in the hepatic-related pathway and down-regulated genes in the leukemic pathway. In addition, discrete groups of tumor cells were found to be associated with oncostatin M signaling, bladder cancer signaling, and paxillin signaling. Interestingly, only trabecular structures and discrete groups of tumor cells were not related to the G2/M DNA damage checkpoint signaling pathway.

**Table 3:** Top 5 Ingenuity Canonical Pathways in different morphological structures of IC NST in comparison with normal breast epithelia

Ingenuity Canonical Pathways	Log10 P value	Ratio	% Down	% Up	Association with other structures (P<0.05)
Tubular structures					
Acute Myeloid Leukemia Signaling	4.8	0.203	11.4	8.9	all str-s
FcγRIIB Signaling in B Lymphocytes	3.9	0.244	12.2	12.2	alv, trab, discr
Role of Macrophages, Fibroblasts and Endothelial Cells in Rheumatoid Arthritis	3.7	0.115	6.4	5.1	all str-s
Molecular Mechanisms of Cancer	3.5	0.107	5.8	4.9	all str-s
Human Embryonic Stem Cell Pluripotency	3.4	0.142	9.0	5.2	all str-s
Alveolar structures					
Regulation of the EMT Pathway	6.3	0.168	9.8	7.1	all str-s
Hepatic Fibrosis / Hepatic Stellate Cell Activation	4.5	0.148	7.7	7.1	all str-s
Role of Macrophages, Fibroblasts and Endothelial Cells in Rheumatoid Arthritis	4.3	0.125	6.4	6.1	all str-s
ErbB Signaling	4.1	0.186	11.6	7.0	all str-s
Actin Cytoskeleton Signaling	4	0.134	8.3	5.1	tub, sol, trab
Solid structures					
Hepatic Fibrosis / Hepatic Stellate Cell Activation	4.8	0.137	6.0	7.7	all str-s
Molecular Mechanisms of Cancer	4.1	0.104	4.9	5.5	all str-s
Role of Macrophages, Fibroblasts and Endothelial Cells in Rheumatoid Arthritis	3.9	0.108	6.1	4.7	all str-s
Cell Cycle: G2/M DNA Damage Checkpoint Regulation	3.6	0.204	0.0	20.4	tub, alv
PAK Signaling	3.6	0.157	12.2	4.9	all str-s
Trabecular structures					
Hepatic Fibrosis / Hepatic Stellate Cell Activation	4.8	0.164	7.1	9.3	all str-s
Fc Epsilon RI Signaling	4.6	0.194	13.0	6.5	tub
Acute Phase Response Signaling	4.2	0.16	8.3	7.7	all str-s
PDGF Signaling	4.0	0.208	15.6	5.2	tub, alv
Acute Myeloid Leukemia Signaling	3.9	0.203	13.9	6.3	all str-s
Discrete groups of tumor cells					
Hepatic Fibrosis / Hepatic Stellate Cell Activation	7.2	0.202	3.8	16.4	all str-s
Acute Myeloid Leukemia Signaling	5.8	0.253	17.7	7.6	all str-s
Oncostatin M Signaling	3.1	0.265	11.8	14.7	trab
Bladder Cancer Signaling	3.0	0.184	9.2	9.2	sol
Paxillin Signaling	2.7	0.168	6.9	9.9	tub, sol, trab

Down: down-regulated genes; Up: up-regulated genes; Tub: tubular; Alv: alveolar; Sol: solid; Trab: trabecular; Discr: discrete; Str-s: structures.

Thus, we found that different morphological structures are characterized the regulation of both common and distinct biological processes and canonical pathways.

### Epithelial and mesenchymal gene expression in different morphological structures of breast tumors

**Figure 4:**
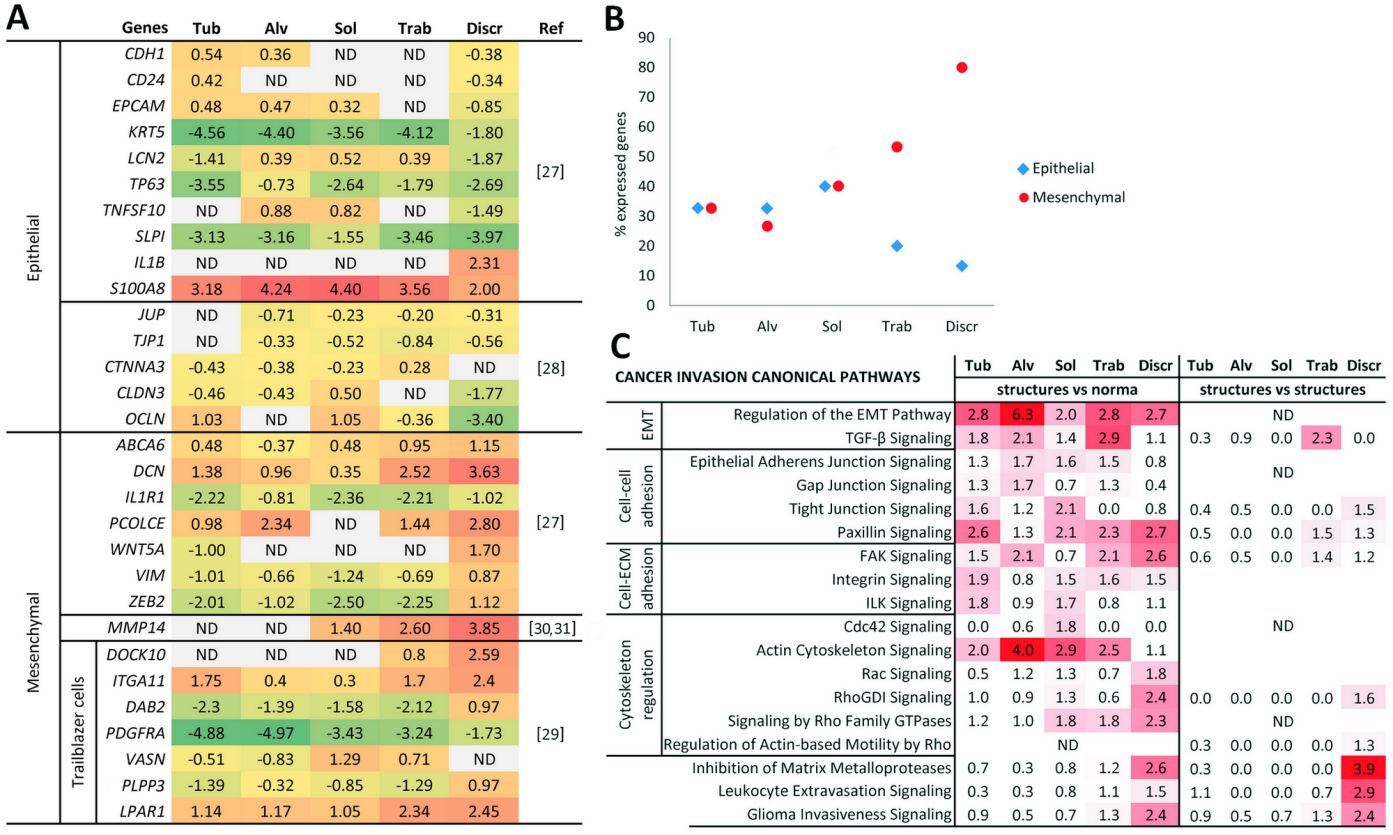
Epithelial-mesenchymal and invasive traits of different morphological structures of IC NST **(A)** The expression levels of genes related to epithelial and mesenchymal cell phenotypes in the structures. The color intensity is proportional to gene expression levels from low (green) to high (red). **(B)** The percentage of expressed epithelial and mesenchymal genes in the structures. **(C)** Cancer invasion pathways enriched in the structures by IPA. Up- and down-regulated transcripts with |log-fold-change| ≥ log_2_1.5 and an unadjusted p value < 0.05 were used for pathway generation. The left panel shows pathways enriched based on the genes that are up- and down-regulated in the structures in comparison with normal breast epithelia, whereas the right panel shows up- and down-regulated genes in comparison of structures amongst themselves (i.e., one any type of structures vs. other structures). The color intensity is proportional to the statistical significance (-log10 (p-value)). The figure was prepared using the data of gene expression microarray profiling of different morphological structures of three BC cases (#1-3 in Supplementary Table 7). Tub: tubular; Alv: alveolar; Sol: solid; Trab: trabecular; Discr: discrete; ND: not determined.

Based on the literature data [27, 28], we chose genes responsible for epithelial and mesenchymal cell phenotypes and checked their array-derived expression levels in the morphological structures (Figure 4A). We also examined the expression of genes, which were described as markers of trailblazer (i.e., leader) cells with mesenchymal traits and were required to initiate collective invasion [29]. Finally, we focused on the MMP14 gene, which was found to be up-regulated in EMT and contribute to tumor cell invasion [30, 31]. Full names of the selected genes and the functions of the encoded proteins are given in Supplementary Table 6.

It turned out that the mesenchymal genes were significantly up-regulated in the discrete groups of tumor cells compared to the other structures (ANOVA test, p**=**0.007). Discrete groups of tumor cells also demonstrated the lowest expression of epithelial markers; however, the differences were not statistically significant (Figure 4A). Other morphological structures demonstrated a different set of up- and down-regulated epithelial and mesenchymal genes (Figure 4A). Counting the number of up-regulated epithelial and mesenchymal genes showed the following patterns. The percentage of active epithelial genes in descending order was as follows: solid, tubular = alveolar, trabecular, and discrete groups. The representation of mesenchymal genes in ascending order was: alveolar, tubular, solid, trabecular, and discrete groups (Figure 4B).

Thus, these data suggest that different morphological structures can represent distinct EMT states, from an epithelial phenotype in tubular, alveolar and solid structures to a mesenchymal phenotype in trabecular and discrete groups of tumor cells.

### Cancer invasion pathways in different morphological structures of breast tumors

The comparison of gene expression profiles relative to normal breast epithelia showed that almost all of the structures possessed signaling pathways involving EMT, intercellular junctions, adhesion between cells and ECM, and cytoskeletal regulation (Figure 4C). In particular, cell-cell adhesions such as epithelial adherens and gap and tight junctions were more pronounced in multicellular arrangements of tumor cells, i.e., tubular, alveolar, solid, and trabecular structures, whereas cell-ECM contacts (i.e., focal and integrin adhesion) were approximately equally in all of the structures. In contrast, discrete groups of tumor cells as well as solid structures were characterized by the significant regulation of cytoskeletal dynamics by the Rho family of GTPases, including Rho and Rac molecules. Interestingly, cytoskeleton regulation via a specific member of Rho family – Cdc42 – was absent in the discrete groups of tumor cells and other structures and was associated only with solid structures. These observations are probably explained by the distinct role of these Rho GTPases in the organization of actin filament assembly [32]. The opposing situation was observed for actin cytoskeletal signaling, which was enriched only in multicellular structures and thus contradicted the aforementioned data regarding the involvement of Rho, Rac, and Cdc42 pathways in discrete groups and solid structures. For example, the detailed analysis of actin cytoskeleton signaling generated from the alveolar structures showed up- and down-regulation of other molecular players (Arp2/3, Myosin, ACTN etc.) together with the absence of Rho, Rac, and Cdc42 activity. Signaling pathways, which are linked with the activity of matrix metalloproteases, transendothelial migration, and cancer invasion in general, were significantly associated with discrete groups of tumor cells and tended to be related to trabecular structures. Moreover, discrete groups of tumor cells showed a more significant association with cancer invasion pathways when transcript expression profiles of the morphological structures were compared with each other without regard to normal breast epithelia (Figure 4C, right panel).

Taken together, these findings once again underscore the association of different morphological structures with distinct EMT states and, as a consequence, with the specific regulation of cancer invasion pathways. In particular, trabecular and discrete groups of tumor cells characterized by a pronounced mesenchymal phenotype demonstrated a significant association with invasion signaling pathways.

### The distribution of CD44^+^CD24^-^ CSCs in different morphological structures of breast tumors

**Figure 5:**
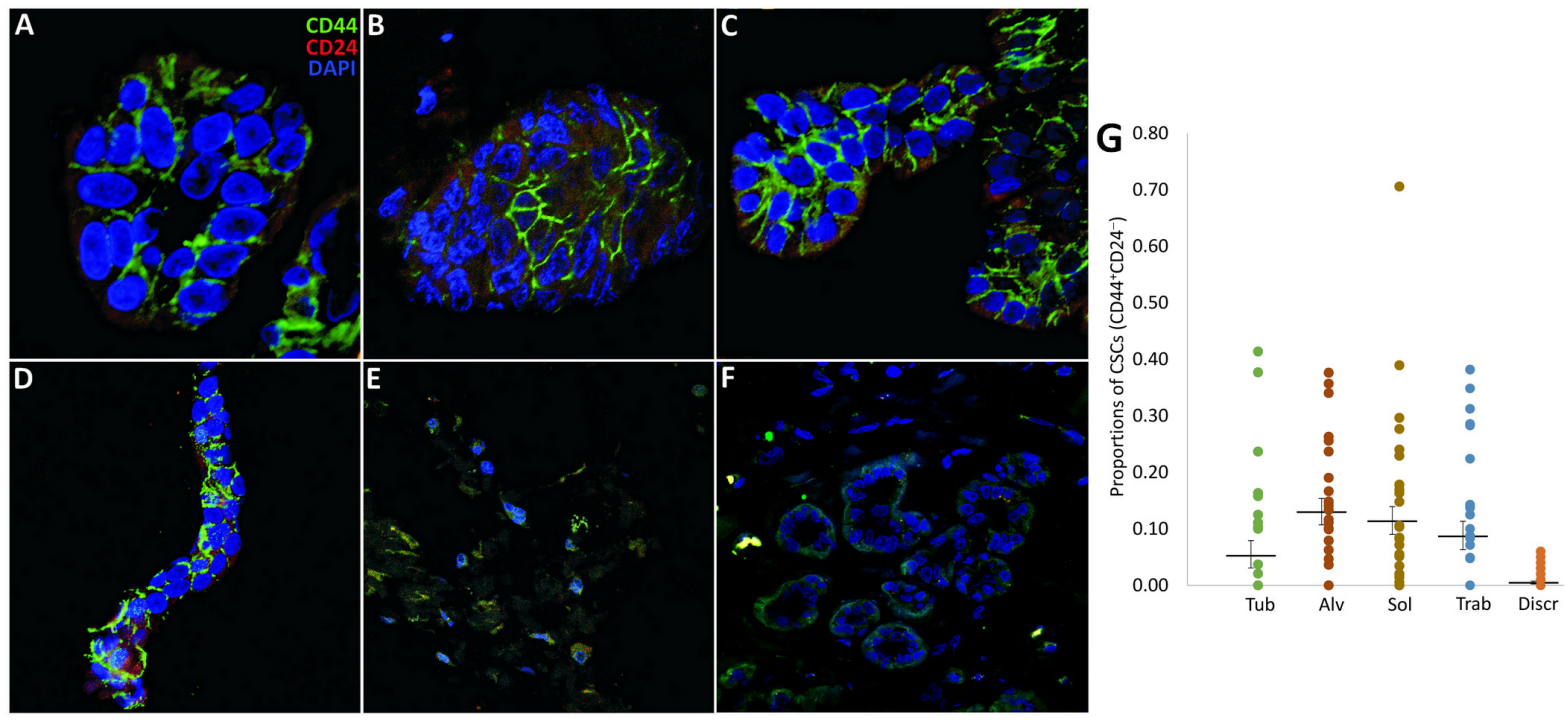
CD44^+^CD24^-^ CSCs in the different morphological structures of IC NST **(A-E)** Representative images of the structures (tubular, alveolar, solid, trabecular, and discrete) immunostained by anti-CD44 and CD24 antibodies. The structures with the abundance of CD44^+^CD24^-^ cells (except the discrete groups) are shown. **(F)** CD44:CD24 staining of adjacent normal breast epithelia (negative control). **(G)** The proportion of CD44^+^CD24^-^ cells in the structures calculated in 36 BC cases. Statistically significant differences in the proportions of CD44^+^CD24^-^ cells were observed in the following comparisons: tubular vs. alveolar, solid, and discrete (Student's t-test, 0.0006<p<0.03), alveolar vs. tubular and discrete (p=0.0006 and 0.00000005, respectively), solid vs. tubular and discrete (p=0.03 and 0.0000001, respectively), trabecular vs. discrete (p=0.00003), and discrete groups vs. other structures (0.00000005<p<0.02). Tub: tubular; Alv: alveolar; Sol: solid; Trab: trabecular; Discr: discrete.

In this part of the study, to verify whether the intratumor morphological heterogeneity of BC is related to stemness, we analyzed the presence of CD44^+^CD24^-^ cells (first described as breast CSCs [33]) in different morphological structures of 36 BC cases. CD44^+^CD24^-^ CSCs were observed in 77.8% (28/36) of luminal breast cancers while the number of these cells was not related on the luminal subtype: A, B HER2^-^, and B HER2^+^ (data not shown). In these patients, CD44^+^CD24^-^ cells were present mainly in solid (96.4% of cases), alveolar (91.6%), trabecular (75.0%), tubular (57.9%), and discrete groups of tumor cells (33.3%). The mean proportion of CD44^+^CD24^-^ cells in the morphological structures was the following: 0.11 (0.09-0.14) in solid, 0.13 (0.11-0.15) in alveolar, 0.09 (0.06-0.11) in trabecular, 0.05 (0.03-0.08) in tubular, and 0.004 (0.002-0.007) in discrete groups (Student's t-test p<0.05: discrete vs. other structures; Figure 5).

Thus, the data obtained indicate that CD44^+^CD24^-^ CSCs predominate in multicellular structures and are almost absent in discrete groups of tumor cells. These results may support the notion [19, 27, 34] that a stem-cell phenotype is much more likely to be associated with co-expression of epithelial and mesenchymal genes which is specific for multicellular structures than with a mesenchymal state found in discrete groups.

## DISCUSSION

During recent years, intratumor heterogeneity has remained one of the hottest topics of modern oncology. Although genetic variability tends to be a more popular subject in the field of intratumor heterogeneity, the importance of morphological diversity and its relationship with cancer prognosis is well-known for a long time [3, 5] and emphasized by recent studies [35–37].

In this study, we focused on the intratumor morphological heterogeneity in IC NST, performed 3D imaging, whole genome copy-number and transcriptome profiling of the different morphological structures of breast tumors and analyzed the distribution of CD44^+^CD24^-^ CSCs. First, the intratumor morphological heterogeneity in BC was not found to be associated with chromosomal abnormalities, which is in agreement with our previous study focused on an aggressive form of BC – invasive micropapillary carcinoma [17]. Second, different morphological structures were shown to represent transcriptionally distinct populations of tumor cells, which are characterized by a specific set of epithelial and mesenchymal genes and the regulation of cancer invasion pathways. Third, morphological structures were found to contain different numbers of CD44^+^CD24^-^ CSCs. Given the present data and the previous results, we described the morphological, cellular and genetic makeup of different morphological structures in IC NST.

Tubular (hollow-like) structures or tube-shaped arrangements of tumor cells are one of the three factors which are considered in the Bloom and Richardson tumor grade [5]. Tubular structures are similar to normal ducts of breast tissue in their shape and are an effective marker for the degree of breast tumor differentiation and for good prognosis [5]. The closeness of tubular structures to normal breast ducts is also confirmed by their lowest proliferative activity. Thus, it is reasonable to presume that these structures demonstrate a pronounced epithelial state. Nevertheless, we found co-expression of epithelial and mesenchymal genes in tubular structures. Probably, some tubular structures demonstrate EMT which results in the formation of their irregular shapes frequently observed in morphological analysis of breast tumors.

Alveolar (morula-like) structures have a rounded (spheroidal) shape and contain up to 30 tumor cells. The presence of alveolar structures in breast tumors is associated with chemoresistance and an increased frequency of lymph node and distant metastasis [8, 11, 14]. Interestingly, the pro-metastatic role of these structures is typical for patients with a poor response to neoadjuvant chemotherapy [14]. Alveolar structures are transcriptionally similar to tubular variants and demonstrate the same degree of “epitheliality”. However, in contrast to tubular and other structures, these structures are characterized by the highest percentage of CD44^+^CD24^-^ CSCs, slightly decreased number of mesenchymal markers, and more considerable association with the EMT pathway. Previously, stemness and EMT were found to correlate with small tumor cell spheroids and emboli described in different cancers [38, 39] and be dimensionally similar to alveolar structures.

Solid structures are represented by shapeless groups of tens and hundreds of tumor cells. At present, there is no clear information regarding the role of solid structures in either the therapeutic response or BC prognosis. Nevertheless, the recent data indicate their chemosensitive role in premenopausal women with BC [14]. Solid structures are also characterized by the same proportion of epithelial and mesenchymal genes and the profound presence of CD44^+^CD24^-^ CSCs. In contrast to tubular and alveolar structures, these morphological variants demonstrate high expression of the *VASN* gene which is known to be enriched in the trailblazer cells [29] and begin to express the *MMP14* gene (MT1-MMP) that is essential for tumor cell invasion [30, 31]. These observations may be related to the fact that many solid structures form protrusions of collectively invading cells.

Trabecular structures are formed by one or two rows of tumor cells and are related to chemoresistance via ABC transporters and an increased frequency of lymph node and distant metastasis [10, 11, 14]. However, in contrast to alveolar structures, the association between trabecular structures and increased metastasis was observed only in cases with a good response to neoadjuvant chemotherapy [14]. Trabecular structures show a high percentage of CD44^+^CD24^-^ cells but are characterized by a dramatic decrease in the expression of epithelial genes and a significant increase in the number of mesenchymal markers. These findings may explain a more significant association of these structures with cancer invasion pathways compared to the tubular, alveolar, and solid structures. Interestingly, only trabecular variants show a 4-fold higher level of the *PKDREJ* gene, which is known to contain point mutations in BC [40]; however, the functions of this gene in tumorigenesis are still unclear.

Discrete groups of tumor cells are represented by single cells and/or arrangements of 2-5 cells and demonstrate a strongly pronounced mesenchymal phenotype, more significant expression of ECM protein genes, the highest association with cancer invasion signaling pathways, and the lowest number of CD44^+^CD24^-^ cells. In addition, discrete groups show the lowest ER and PR expression that can be also an indicator of EMT as found previously [41]. Surprisingly, these groups of tumor cells, like trabecular structures, are characterized by the highest proliferative activity. Discrete groups of tumor cells were recently found to be related to lymph node and distant metastasis [14]. In other studies, tumor buds, which are defined as single cancer cells or clusters with fewer than five cancer cells at the invasive front of the tumor and thus morphologically similar with discrete groups, were also associated with worse disease-free and overall survival in invasive BC [42]. Similar to discrete groups, tumor buds have been described as tumor cells demonstrated reduced epithelial and increased mesenchymal features and associated with the hybrid EMT phenotype displaying collective cell migration [42]. Nevertheless, it is reasonable to presume that discrete groups as well as tumor buds represent the heterogeneous population among which isolated tumor cells and small clusters can demonstrate different EMT states (from hybrid to mesenchymal). Moreover, single tumor cells themselves are highly heterogeneous and represented by genetically and phenotypically distinct subpopulations that can evolve from a common ancestor [43]. This point is supported by our data that these structures display an extended and extremely diverse list of specific over- or underexpressed genes. Meanwhile, discrete groups of tumor cells isolated from three regions of one breast tumor (case 3) probably shared the origin-specific duplication of the *PAX6* gene. Thus, it is reasonable to suggest that further studies should be focused on the identification of specific subpopulations of discrete groups of tumor cells and the investigation of their phenotype and association with BC progression and prognosis.

**Figure 6:**
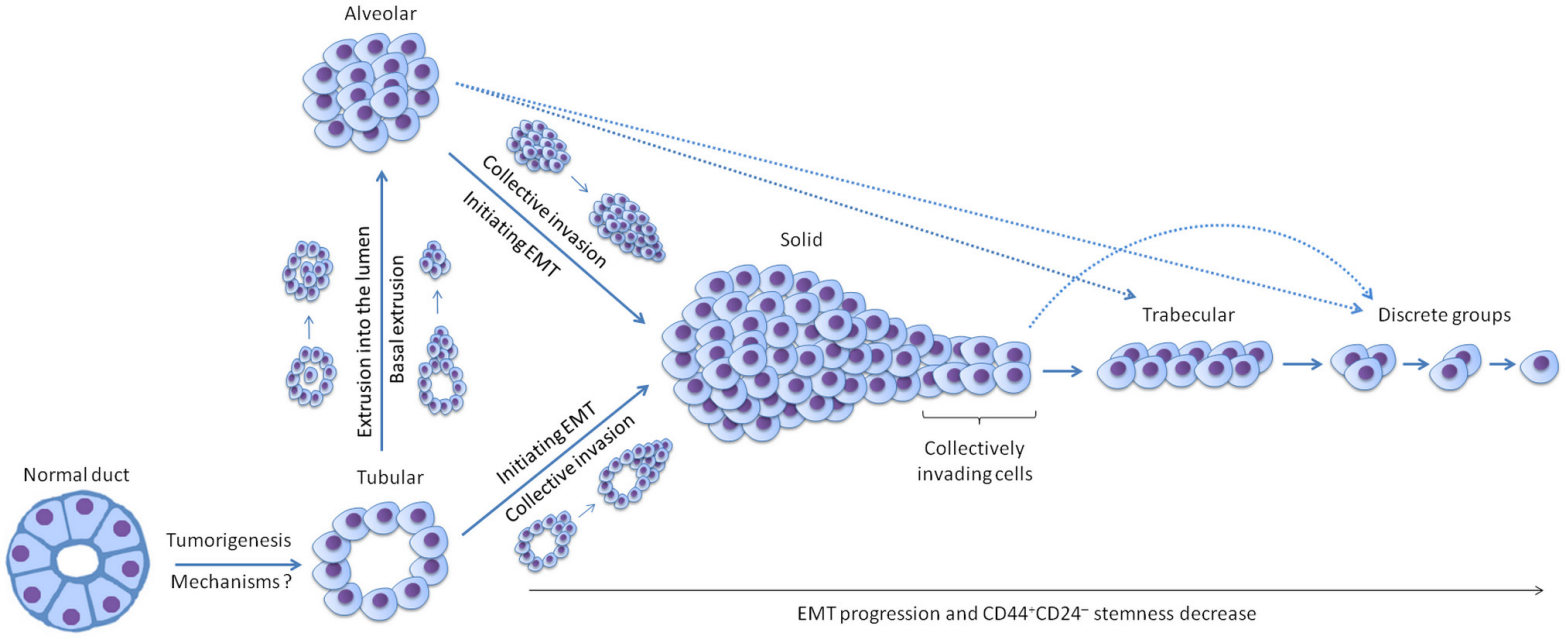
Hypothetical model of the origin and evolution of different morphological structures in breast cancer The model is based on the data of gene expression microarray profiling indicating that transcriptional differences including the number of mesenchymal genes are increased in ascending order: tubular = alveolar, solid, trabecular, and discrete groups. Tubular structures are probably formed first from normal breast ducts during tumorigenesis; however, the underlying mechanisms are unknown. This suggestion is confirmed by a high differentiation of tubular structures that manifested in their closeness to breast ducts in the shape and their lowest proliferative activity. Alveolar structures originate from tubular structures via basal extrusion of groups of cells into ECM and/or extrusion of single tumor cells into the lumen followed by their proliferation and growth. This assumption is supported by the transcriptional similarity of these two types of structures which could be arisen only during the process (e.g. extrusion) that does not globally change a cell transcriptome. Solid structures are probably derived both from tubular and alveolar structures through initiating EMT and the formation of the front of collectively invading cells. This front can be detached from the main solid mass and transform into trabecular structures via the progression of the EMT program, collective migration, and a decrease in cell-cell adhesions. The completion of the EMT process results in the disruption of cell-cell adhesions and the formation of small groups of tumor cells and single tumor cells covered by the umbrella term “discrete groups of tumor cells”. Overall, the progression of the EMT program results in changes in CD44^+^CD24^–^ stemness of different morphological structures. The proposed model is preliminary and does not yet consider other possible ways of the evolution of different morphological structures. In particular, it is not understood if trabecular structures can originate from alveolar structures and discrete groups – directly from solid and alveolar structures (these possible transformations are marked by broken lines).

Based on the above discussion, we hypothesize that the development of morphological structures represents a plastic and complex process that proceeds via EMT and/or stemness and is controlled by a variety of external and internal factors. For example, this morphogenesis can be stimulated by macrophages and fibroblasts that infiltrate breast tumors [12]. In addition, epithelial extrusion comprehensively described by Rosenblatt's group [44, 45] may contribute to the morphogenesis by generating single tumor cells/groups of cells and their subsequent transformation into different morphological structures. To support these assumptions, we propose a model of the origin and evolution of different morphological structures in BC (Figure 6). However, this model has several shortcomings and should be supplemented with additional studies. For example, various hierarchical relationships between morphological structures in the number of common chromosomal aberrations found in this study may indicate the possibility of shifting from one structure to another in a stochastic manner. In addition, our previous study shows that any type of structures is equally probable in breast tumors with one type of structure, whereas cases with two types of structures are more likely to contain trabecular structures [9].

Taken together, our findings show that different morphological structures in BC are represented by transcriptionally distinct tumor populations with the specific 3D organization, varied degrees of EMT and stemness, and the regulation of specific signaling pathways. The assessment of morphological diversity within breast tumors may provide valuable information regarding the EMT degree and thereby predict cancer prognosis as found in our previous studies [8, 11, 14]. Moreover, intratumor morphological heterogeneity may represent an attractive model for the understanding of BC invasion.

## MATERIALS AND METHODS

### Patients and specimens

Patients (n=40) with luminal A and B IC NST (*T_1-3_N_0-3_M_0-1_*), between 36 and 68 years of age (mean age: 54.4±9.50), and treated in the Cancer Research Institute, Tomsk NRMC (Tomsk, Russia) between 2012 and 2015 were included (Supplementary Table 7). IC NST was defined according to the World Health Organization’s recommendations [7]. The inclusion of luminal subtypes of IC NST was motivated by the fact that these breast cancers demonstrate more pronounced intratumor morphological heterogeneity than HER2-positive and triple-negative forms [9]. All cases were without any preoperative therapy and underwent surgery (radical resection or sectoral resection or mastectomy). After surgery, adjuvant chemotherapy or hormonal therapy was given.

The formalin-fixed, paraffin-embedded (FFPE) samples were used for 3D imaging (a total of 3 samples) and immunofluorescence analysis (n=40, Supplementary Table 8). The frozen tumor and normal tissue specimens were used for a laser microdissection-assisted array comparative genome hybridization (aCGH, n=3), gene expression microarrays (n=3), and qRT-PCR (n=7, Supplementary Table 8).

The procedures followed in this study were in accordance with the Helsinki Declaration (1964, amended in 1975 and 1983). This study was approved by the institutional review board, all patients signed an informed consent for voluntary participation, and the number of ethical approval was 10 (29 September 2011).

### 3D imaging

Forty μm-thick sections were prepared from FFPE tumor samples, deparaffinized, rehydrated, processed for heat-induced epitope retrieval in PT Link (Dako, Denmark) with EDTA buffer (pH 8.0), and blocked with 3% bovine serum albumin (Amresco, USA) in PBS. The sections were incubated with the primary antibody against cytokeratin 7 (ready-to-use, clone OV-TL, Dako, Denmark) followed by incubation with rhodamine-conjugated anti-mouse IgG (H+L) (Invitrogen, USA). Finally, Vectashield mounting medium (Vector Laboratories, USA) containing DAPI was used to detect nuclei and mount the specimens. Morphological structures were visualized using the rhodamine channel of a LSM 780 NLO confocal microscope (Carl Zeiss, Germany) with a 63 plan apochromat objective, numerical aperture (z1), and immersion oil. A series of 40 to 90 z-sections were taken at 0.2-0.5 μm distance. Images were produced with ZEN-2012-SP1 (black edition, version 8.1) software (Carl Zeiss, Germany).

### Immunohistochemistry

IHC was applied to assess ER, PR, HER2, and Ki-67 expression in breast tumors. The following antibodies were used: anti-ER (clone 1D5, mouse, monoclonal, ready-to-use, Dako, Denmark), anti-PR (clone PgR636, mouse, monoclonal, ready-to-use, Dako, Denmark), anti-HER2 (A0485, rabbit, polyclonal, 1:500, Dako, Denmark), and anti-Ki-67 (clone MIB-1, mouse, monoclonal, ready-to-use, Dako, Denmark). IHC was performed as previously described [9].

The ER and PR immunostaining was scored using the HSCORE method [46]. The HER2 status was assessed on a scale 0-3+. Tumors with immunohistochemical scores of 3+ and 2+ (confirmed by FISH) were interpreted as positive and with scores of 0 or 1+ as negative for HER2 overexpression (HER2^+^). Ki-67 expression was calculated as a percentage of positively stained cells. At least 10 view fields on 1000 cells at 400x magnification were analyzed per sample. IHC data was used to classify breast tumors into different molecular subtypes and to analyze differences in the hormonal receptor status, HER2 and Ki-67 expression between different morphological structures in breast tumors. Molecular subtypes of IC NST were categorized according St Gallen recommendations [47]: luminal A (ER^+^ and/or PR^+^, HER2^-^, and Ki-67 < 20%), luminal B HER2^-^ (ER^+^ and/or PR^+^, HER2^-^, and Ki-67 ≥ 20%), and luminal B HER2^+^ (ER^+^ and/or PR^+^, HER2^+^).

### Laser microdissection

Tubular, solid, alveolar, trabecular structures, and discrete groups of tumor cells (Supplementary Figure 2) were isolated from hematoxylin and eosin stained five μm-thick sections of frozen tumor samples using a PALM MicroBeam laser capture microdissection (Carl Zeiss, Germany) as described previously [10, 17]. All sections were reviewed by two pathologists (MVZ and SVV) who are competent and experienced in the field of BC pathology. The following criteria were used to identify different morphological structures. Tubular structures were identified as rows of tube-shaped cell aggregations. Alveolar structures were represented by round-shaped groups of 10-30 cells. Solid structures were identified as groups comprising of hundreds of tumor cells. Trabecular structures were represented by one or two rows of tumor cells. Discrete groups of tumor cells were detected as single cells or as groups of up to five cells. It is important to note that tumors from different patients had different types of morphological structures (Supplementary Table 7).

In a case of analysis of chromosomal aberrations, three distinct samples (regions) of each breast tumor (n=3) were laser microdissected. Five types of morphological structures were obtained from each tumor region: 2-3 samples (∼20-30 cells) of tubular, alveolar, and trabecular structures, one sample (∼70-80 cells) of solid structure, and 10 samples (up to 20 cells) of discrete groups of tumor cells (Supplementary Table 8). The microdissected material (a total of 45 samples) was collected in adhesive caps (Carl Zeiss, Germany) and stored at room temperature until whole genome amplification. In a case of expression analysis, five types of morphological structures were isolated from all three samples of each breast tumor (n=10): tubular, alveolar, and trabecular structures in the number of 90-120 samples (∼900-1500 cells), solid structures – 50-60 samples (up to 5000 cells), and discrete groups of tumor cells – 300-350 samples (∼400-600 cells). In addition, histologically normal breast epithelia (90-120 ducts, or ∼900-1500 cells) were microdissected from 10 samples of tumor-adjacent normal tissue (Supplementary Table 8). The number of microdissected material varied from case to case in the aforementioned range and depended on the size of samples of tumor/normal tissue and representation of structures/normal ducts. It must be noted that seven cases contained all types of structures in breast tumors, whereas tumors of three patients lacked tubular structures. The microdissected material (n=57) was collected in RLT lysis buffer (Qiagen, USA) and stored at -80°C until RNA isolation. The differences in the number of microdissected samples (cells) were due to the requirements of whole genome and transcriptome amplification protocols used in this study.

### aCGH

The microdissected material (n=45) as well as human female DNA (Agilent, USA) were used to perform whole genome amplification using the PicoPLEX WGA Kit (Rubicon, USA). Analysis of chromosome aberrations was performed using the SurePrint G3 Unrestricted CGH ISCA v2, 8x60K microarrays (Agilent, USA). Sample preparation was carried out using the SureTag DNA Labeling Kit (Agilent, USA). The scanning was performed using a SureScan Microarray Scanner. Data analysis was conducted using Cytogenomics Software (v. 3.0.2.11; Agilent, USA). The microarray data have been submitted to GEO (Gene Expression Omnibus, accession number GSE80758). The whole analysis code used in this work is given in the Supplementary Data (aCGH.R).

### Gene expression microarrays

Total RNA was extracted from the microdissected samples (n=18) using the RNeasy Plus Micro Kit (Qiagen, USA). RIN varied from 2.5 to 7.7 (average ∼5.6). RNA samples were amplified using the Ovation PicoSL WTA System V2 kit (NuGEN, USA). Transcriptome profiling was carried out using the SurePrint G3 Human GE v2, 8x60K microarrays (Agilent, USA). cDNA samples were Cy3-labeled using the SureTag DNA labeling kit (Agilent, USA). Hybridization to microarray slides, subsequent washing and drying of the slides were performed according to the Agilent hybridization protocol with the following modifications: 2 μg of the labeled cDNA was hybridized for 22 h at 65°C and the cDNA was not fragmented before hybridization. The scanning was conducted using a SureScan Microarray Scanner and signals were extracted using Feature Extraction software v. 10.7.3.1 (Agilent, USA). Stored data were evaluated using the R software (R Development Core Team, 2008) and the limma package from BioConductor [48]. Log mean spot signals were taken for further analysis. Expression levels were normalized to normal breast epithelia permitting the identification of pathogenetically relevant genes. Transcripts were ranked for differential expression using a moderated t-statistic as implemented in the limma package. The microarray data have been submitted to GEO (Gene Expression Omnibus, accession number GSE80754). All the data analysis code used to generate the results of gene expression microarrays is shown in the Supplementary Data (GEM.R).

### qRT-PCR

Gene expression data generated by microarrays were validated using qRT-PCR analysis of 6 unlinked genes (Supplementary Table 9) in different morphological structures of breast tumors of 7 patients (Supplementary Table 8). The microdissected samples (n=39) were used for RNA isolation by the RNeasy Plus Micro Kit (Qiagen, USA). *RNA samples were amplified using the* QuantiTect Whole Transcriptome Kit (Qiagen, USA). qRT-PCR was conducted based on TaqMan technology using a Rotor-Gene-6000 instrument (Corbett Research, Australia) and performed in triplicate reactions. PCR conditions, the temperature profile, and the algorithm for calculating expression levels are given in our previous study [10]. The results were presented as log2 fold-changes in the expression of the gene of interest relative to housekeeping genes (*ACTB1* and *GAPDH*) and normal breast epithelia.

### Immunofluorescence analysis

Seven μm-thick sections were prepared from FFPE tumor samples (n=36), deparaffinized, rehydrated, processed for heat-induced epitope retrieval in PT Link (Dako, Denmark) with EDTA buffer (pH 8.0), and blocked with 3% bovine serum albumin (Amresco, USA) in PBS. Subsequently, the sections were incubated with the primary antibody against CD44 (1:50, v6 clone VFF7, Leica Biosystems, Germany) followed by incubation with Alexa fluor 488-conjugated anti-mouse IgG (H+L) (Invitrogen, USA) and the primary conjugated antibody against CD24 (1:50, SE3 clone, NovusBio, USA). Finally, Vectashield mounting medium (Vector Laboratories, USA) containing DAPI was used to detect nuclei and mount the specimens. The samples were analyzed using a LSM 780 NLO confocal microscope (Carl Zeiss, Germany). At least 10 structures of each type (tubular, alveolar, solid, and trabecular) and 100 discrete groups were counted per sample. Only tumor cells with CD44 membrane staining without membrane and cytoplasm localization or colocalization of CD24 were defined as CSCs (Figure 5). Two parameters were considered. First, we determined the frequency of CD44^+^CD24^-^ cells, particularly which morphological structures predominantly contained these cells. Second, we calculated the percentage of CD44^+^CD24^-^ cells, which meant the proportion of these cells regarding all tumor cells in each type of morphological structures. Mean proportions of CD44^+^CD24^-^ cells were estimated using the arcsine-transformed data. Figures given in the text are the mean estimates; values in brackets represent the range corresponding to the range of the mean plus and minus one standard error of the arcsine-transformed values. Adjacent normal breast tissue was used as a negative control (Figure 5). Positive CD24 staining, particularly CD44^+^CD24^+^ cells, is shown in Supplementary Figure 3.

### Functional enrichment, pathway and statistical analyses

Up- and down-regulated transcripts with |log-fold-change| ≥ log_2_1.5 and an unadjusted P value < 0.05 were used in functional enrichment and pathway analysis. The identification of shared and specific genes was carried out by Venn diagram analysis using Draw Venn (http://bioinformatics.psb.ugent.be/webtools/Venn/). The gene ontology (GO) analysis was performed by Enrichr [49]. A Benjamini-Hochberg adjusted P value < 0.05 (Fisher exact test) was used as a threshold to consider GO biological processes as being significantly enriched. REVIGO was used to summarize and visualize GO terms [50]. Canonical pathways were generated through the use of QIAGEN’s Ingenuity Pathway Analysis (IPA, QIAGEN Redwood City, www.qiagen.com/ingenuity), and the results were discussed using the following values: -log10(p-value), or a negative log of the p-value derived from the Fisher’s Exact test, ratio (the percentage of genes involved in the canonical pathway), and percentage of up- and down-regulated genes.

The association of different morphological structures with chromosomal aberrations was assessed using a hierarchical cluster analysis of common chromosome aberrations by calculating the Euclidean distance metric with complete linkage. Gene expression between different morphological structures was compared by ANOVA test. The correlation between gene expression data generated by microarrays and qRT-PCR was carried out using a linear regression analysis. Differences in the proportions of CD44^+^CD24^-^ CSCs between morphological structures were analyzed by Student's t-test. All statistical procedures were carried out using STATISTICA 8.0 (Statsoft Inc., USA).

## SUPPLEMENTARY MATERIALS FIGURES, VIDEOS AND TABLES



























## Abbreviations

BC: breast cancer
CSCs: cancer stem cells
ECM: extracellular matrix
EMT: epithelial-mesenchymal transition
ER: estrogen receptors
FDR: false discovery rate
FFPE: formalin-fixed, paraffin-embedded
IC NST: invasive carcinoma of no special type
IHC: immunohistochemistry
PR: progesterone receptors
qRT-PCR: quantitative reverse transcription PCR
