# Supplementary material for: Clinically relevant morphological structures in breast cancer represent transcriptionally distinct tumor cell populations with varied degrees of epithelial-mesenchymal transition and CD44+CD24- stemness
Source: Oncotarget. 2017 May 19;8(37):61163–80. doi: 10.18632/oncotarget.18022 (PMC5617414; doi:10.18632/oncotarget.18022)
Supplement: Supplementary file 1 [file oncotarget-08-61163-s001.pdf]

Clinically relevant morphological structures in breast cancer represent transcriptionally distinct tumor cell populations with varied degrees of epithelial-mesenchymal transition and CD44⁺CD24⁻ stemness

SUPPLEMENTARY MATERIALS

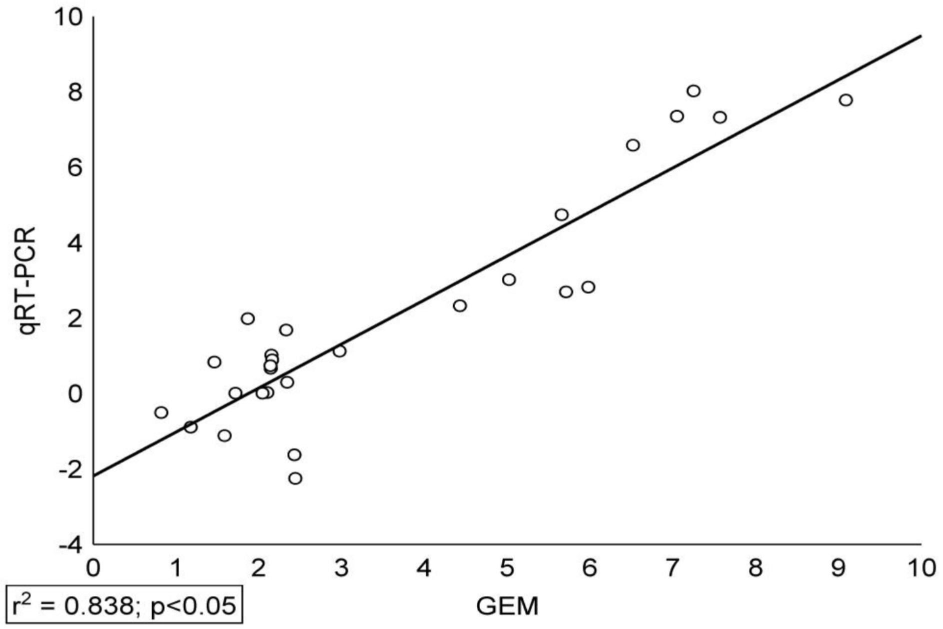

Supplementary Figure 1: Correlation between gene expression microarrays and qRT-PCR. Plot shows the log mean spot signals and the log-transformed expression levels of 6 unlinked genes detected by gene expression microarrays (GEM, x-axis) and qRT-PCR (y-axis), respectively.

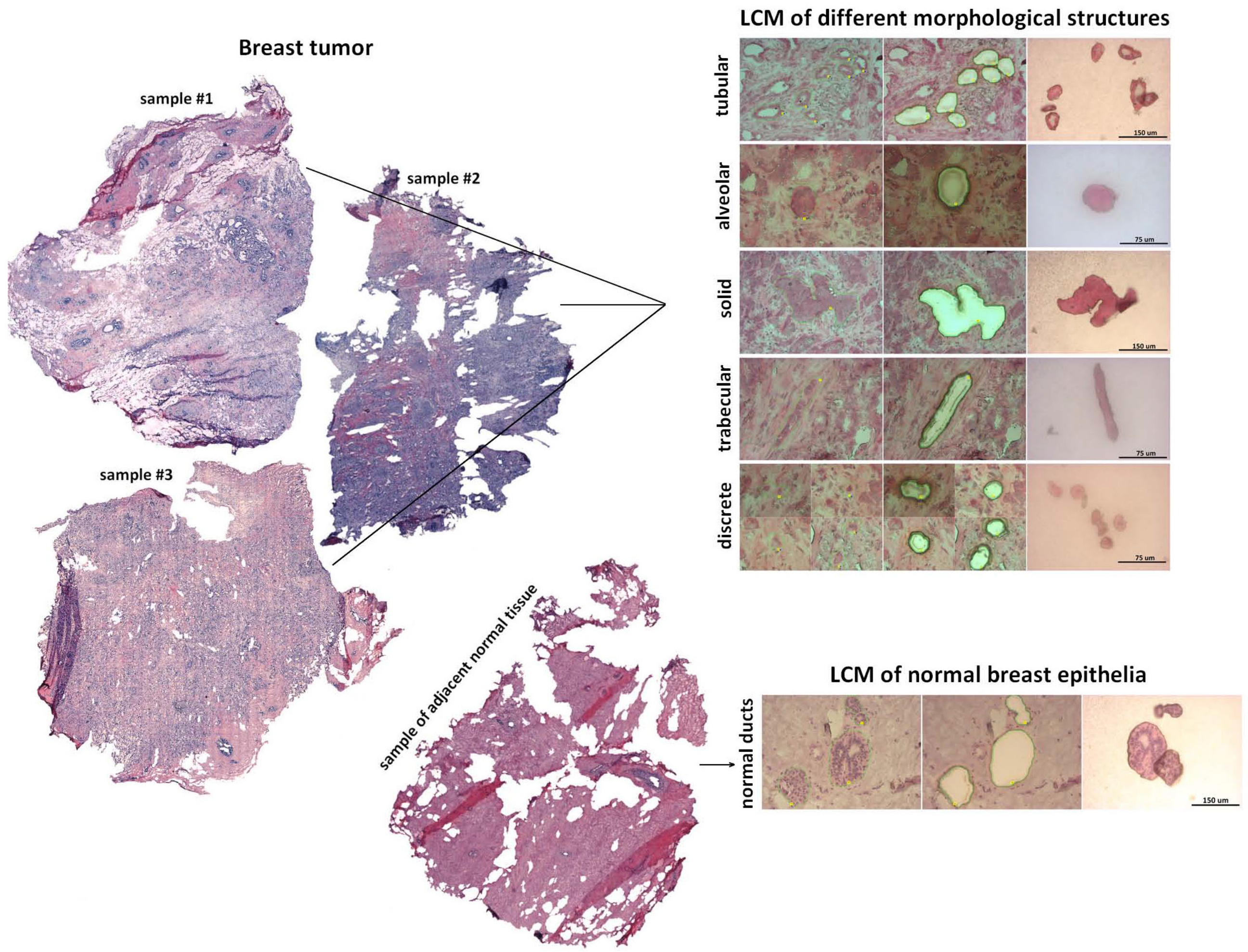

Supplementary Figure 2: Laser microdissection (LCM) of different morphological structures and normal breast epithelia. Tubular, alveolar, solid, trabecular structures, and discrete groups of tumor cells were isolated from hematoxylin and eosin stained sections of breast tumor specimens. In a case of aCGH analysis, three distinct samples (regions) of each breast tumor (n=3) were laser microdissected. Five types of morphological structures were obtained from each tumor region. In a case of expression analysis, five types of morphological structures were isolated from all three samples of each breast tumor and normal breast epithelia (ducts) – from the sample of tumor-adjacent tissue (a total of 10 cases).

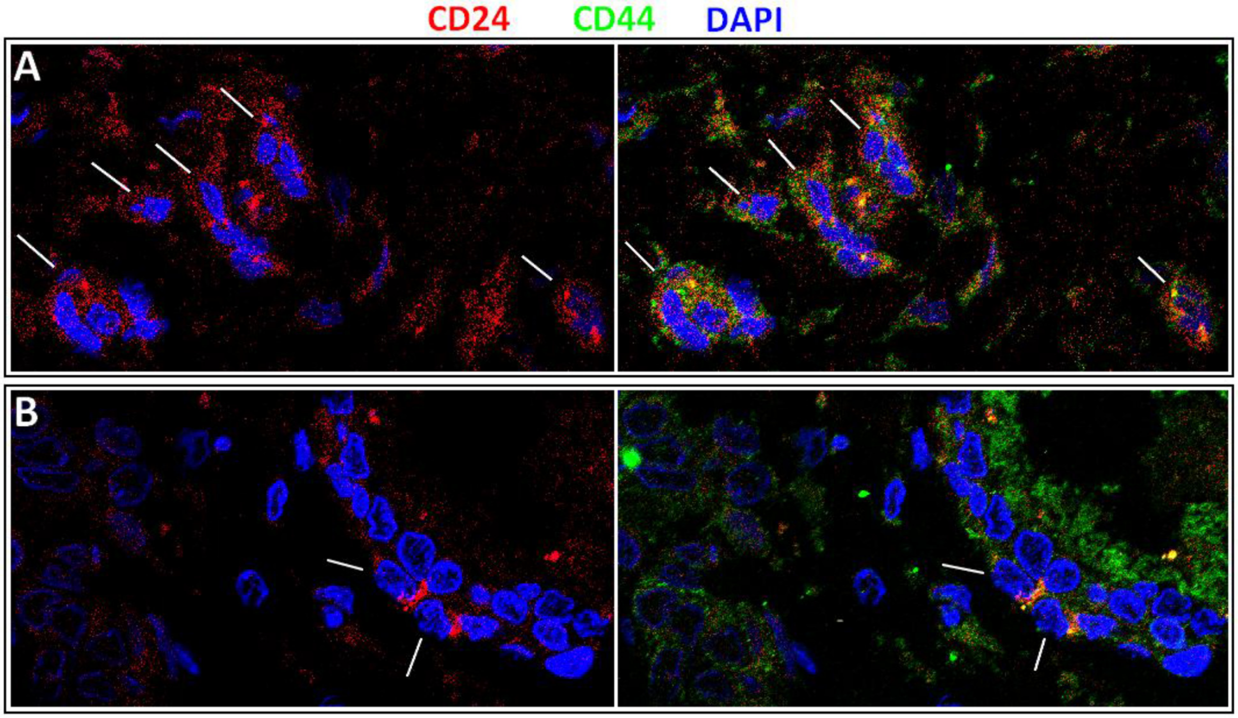

Supplementary Figure 3: CD24 expression in two different breast tumors (A, B). CD24-positive cells are marked by arrows.

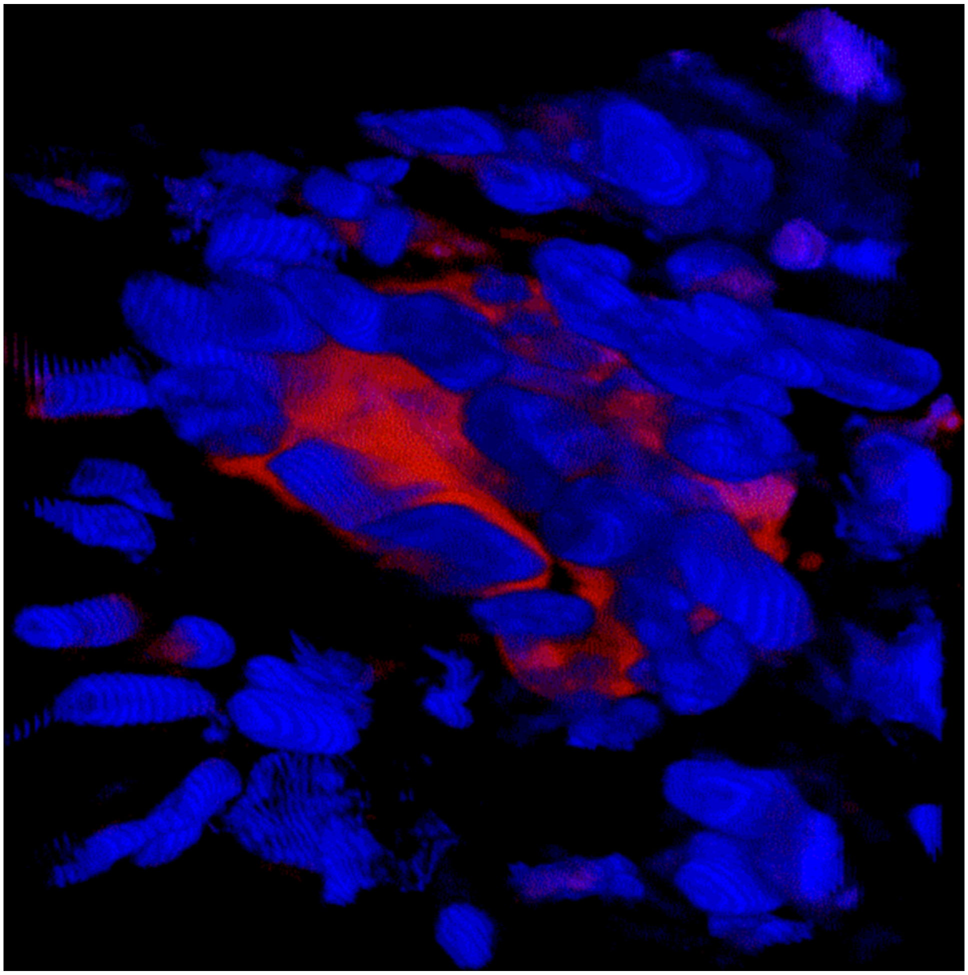

Supplementary Movie 1: 3D movie of tubular structure.

See Supplementary Movie 1

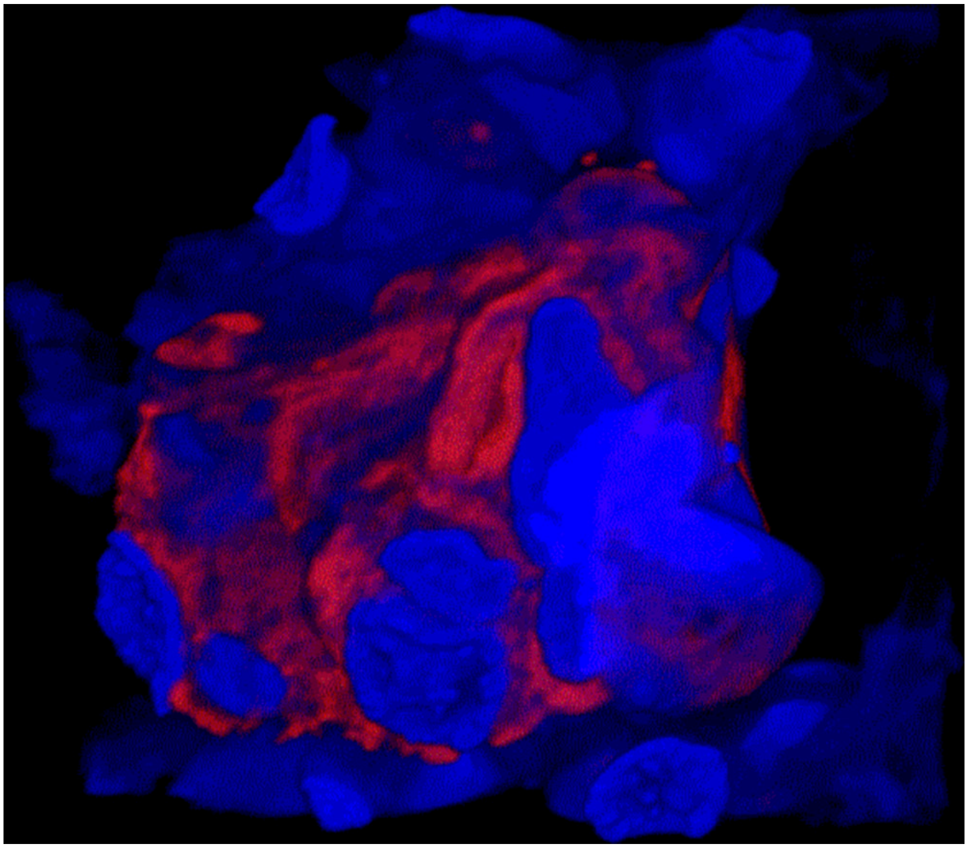

Supplementary Movie 2: 3D movie of alveolar structure.

See Supplementary Movie 2

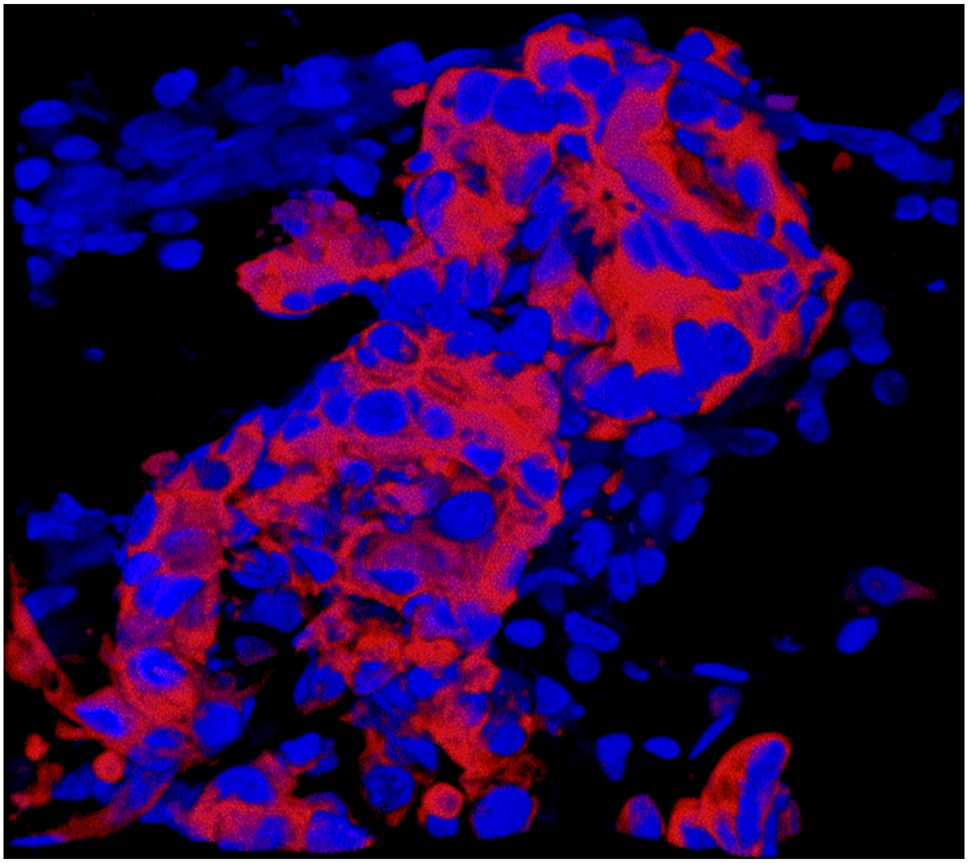

Supplementary Movie 3: 3D movie of solid structure.

See Supplementary Movie 3

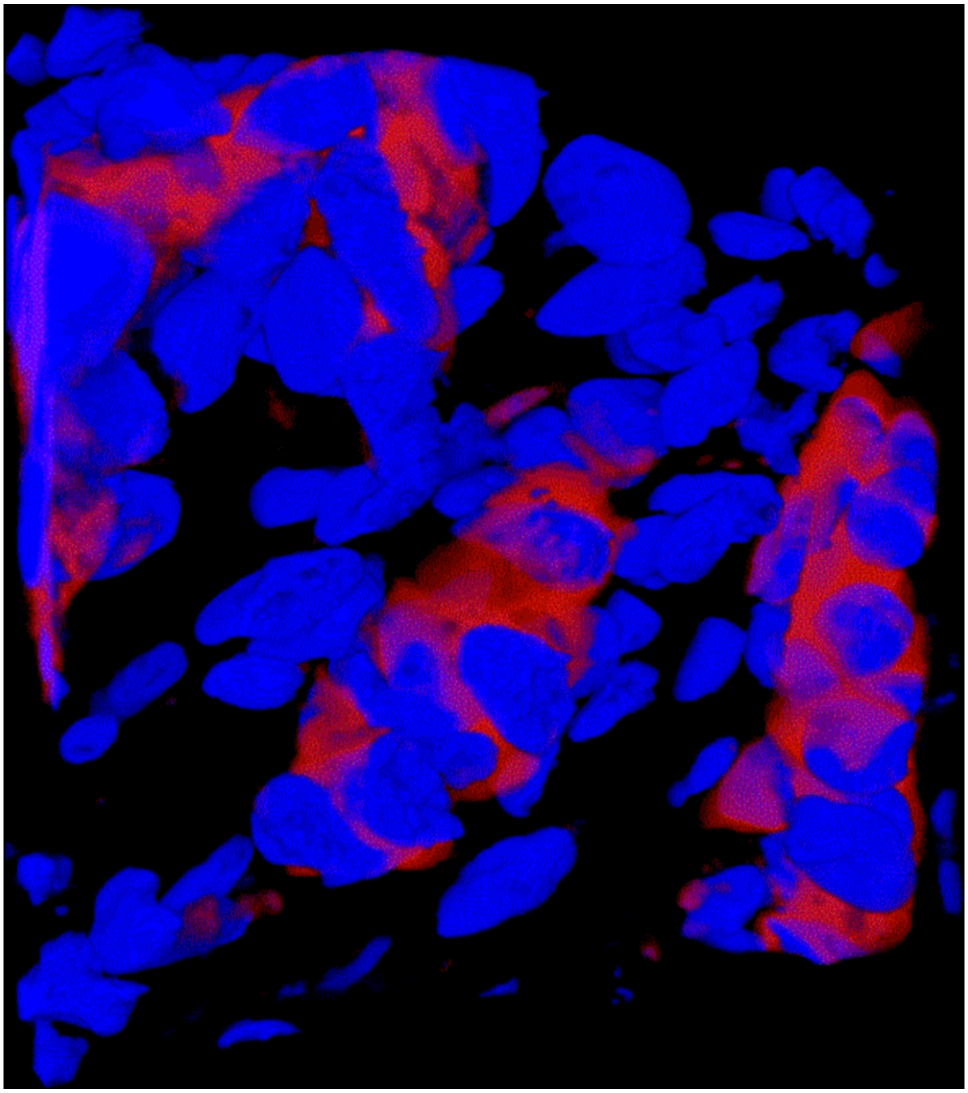

Supplementary Movie 4: 3D movie of trabecular structure.

See Supplementary Movie 4

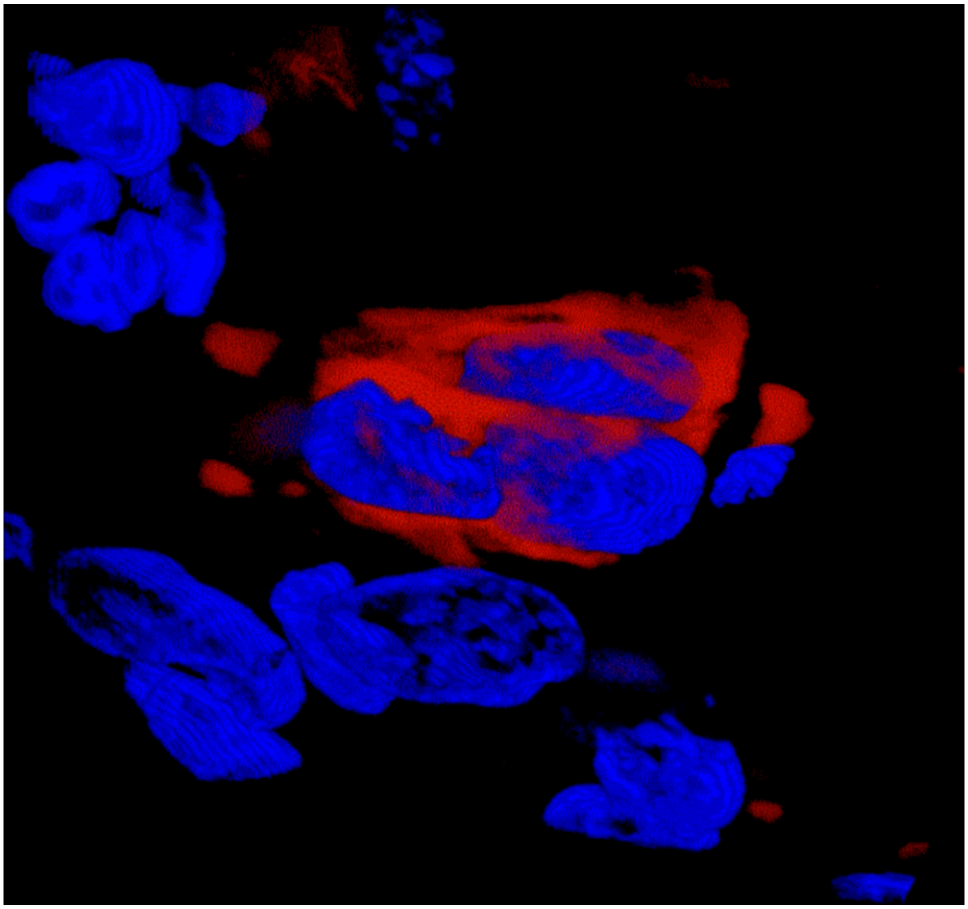

Supplementary Movie 5: 3D movie of small group of tumor cells.

See Supplementary Movie 5

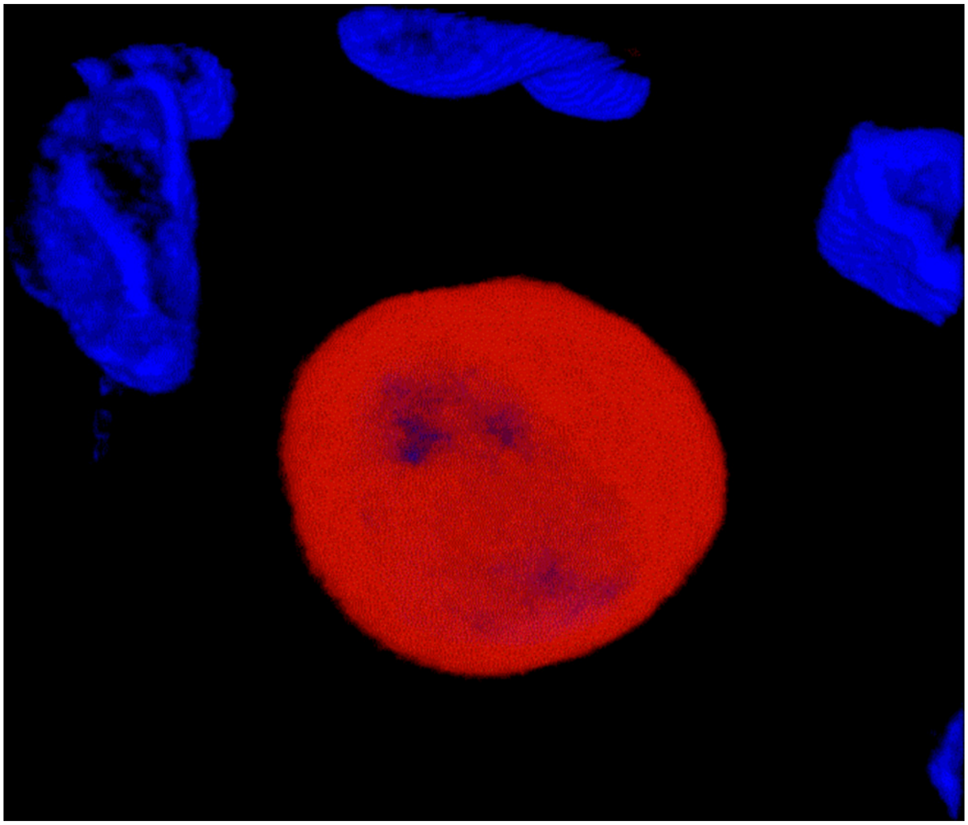

Supplementary Movie 6: 3D movie of single tumor cell.

See Supplementary Movie 6

Supplementary Table 1: The percentage of tumor cells expressing hormonal receptors and Ki-67 in different morphological structures in breast tumors

	Tubular	Alveolar	Solid	Trabecular	Discrete
	**1**	**2**	**3**	**4**	**5**
Estrogen receptors	82.8±13.6	76.8±25.6	86.4±21.9	74.9±24.9	71.7±25.2<br>p_{5-3}=0.05
Progesterone receptors	78.5±29.7	70.6±32.1	64.6±37.5	62.4±33.3	48.3±43.1<br>p_{5-1}=0.02<br>p_{5-2}=0.04
Ki-67	2.1±2.3<br>p_{1-3}=0.05<br>p_{1-4}=0.08<br>p_{1-5}=0.01	6.4±10.9	6.8±9.2	11.2±20.9	11.4±19.8

ER, PR, and Ki-67 protein expression was analyzed using immunohistochemistry. P values demonstrate the significance of differences in the expression of markers between different morphological structures: p_{1-2}, between tubular and alveolar, p_{1-3}, between tubular and solid, etc. Only $p<0.1$ are shown.

Supplementary Table 2: The frequency of HER2 expression in different morphological structures in breast tumors

HER2 scoring	% (n/N)				
	Tubular	Alveolar	Solid	Trabecular	Discrete
0	42.9 (3/7)	27.3 (6/22)	41.7 (5/12)	23.8 (5/21)	31.3 (5/16)
1+	57.1 (4/7)	54.5 (12/22)	33.3 (4/12)	57.1 (12/21)	56.3 (9/16)
2+	ND	13.6 (3/22)	8.3 (1/12)	14.3 (3/21)	6.3 (1/16)
3+	ND	4.5 (1/22)	16.7 (2/12)	4.8 (1/21)	6.3 (1/16)

HER2 protein expression was analyzed using immunohistochemistry and calculated on a scale 0-3+ according to the ASCO/CAP guidelines (Wolff et al., Arch Pathol Lab Med 2007), where 0 or 1+: no staining or weak, incomplete membrane staining in any proportion of tumor cells; 2+ – strong, complete membrane staining in less than 30% of tumor cells or weak/moderate heterogeneous complete staining in more than 10% of tumor cells; 3+ – uniform, intense membrane staining in >30% of tumor cells.

%, the percentage of HER2 expression (from 0 to 3+); n, the number of cases with HER2 expression in different morphological structures; N, the total number of breast cancers demonstrating different morphological structures.

Supplementary Table 3: The list of transcripts expressed in different morphological structures at $p<0.05$ (FDR-corrected)

See Supplementary File 1

Supplementary Table 4: GO enrichment analysis of up-regulated transcripts overlapping between different morphological structures of breast tumors (adjusted p value < 0.05)

See Supplementary File 2

Supplementary Table 5: GO enrichment analysis of down-regulated transcripts overlapping between different morphological structures of breast tumors (adjusted p value < 0.05)

See Supplementary File 3

Supplementary Table 6: The characterization of genes used for the analysis of epithelial and mesenchymal features in different morphological structures of breast tumors

See Supplementary File 4

Supplementary Table 7: Clinicopathological characteristics of breast cancer patients

See Supplementary File 5

Supplementary Table 8: The number of patients and samples used in this study

	3D imaging	aCGH		Gene expression microarrays	qRT-PCR	IHC	Immunofluorescence staining
No. of patients/ tumors (total group, n=40, Table S1)		3 (cases #1,2,3)			7 (cases #4-10)	23 (cases #1-8,11,16-18,20-23,25-27,30,32,34-35)	36 (cases #1,4-8,11-40)
No. of analyzed/ isolated morphological structures from each tumor:							
tubular	1	2-3 (20-30 cells)	from each of three regions of each tumor	90-120 (900-1500 cells)	from any region of each tumor	at least 10 view fields on 1000 cells	at least 10
alveolar	1	2-3 (20-30 cells)		90-120 (900-1500 cells)			at least 10
solid	1	1 (70-80 cells)		50-60 (up to 5000 cells)			at least 10
trabecular	1	2-3 (20-30 cells)		90-120 (900-1500 cells)			at least 10
discrete	1	10 (up to 20 cells)		300-350 (400-600 cells)			at least 300
normal breast ducts	NA	NA		90-120 (900-1500 cells)		NA	NA
No. of DNA/RNA samples of each structure:							
tubular	NA	9		3	4*	NA	NA
alveolar		9		3	7		
solid		9		3	7		
trabecular		9		3	7		
discrete		9		3	7		
normal breast ducts		NA		3	7		
Total no. of DNA/ RNA samples	NA	45		57		NA	NA

IHC: immunohistochemistry; aCGH: array comparative genomic hybridization; qRT-PCR: quantitative reverse transcription PCR; NA: not applicable; *, three cases did not have tubular structures in tumors. The approximate number of tumor cells of isolated morphological structures by laser microdissection is shown in the table.

Supplementary Table 9: The comparison of gene expression levels obtained by gene expression microarrays and qRT-PCR

See Supplementary File 6
