# Supplementary figures and images for: Clinically relevant morphological structures in breast cancer represent transcriptionally distinct tumor cell populations with varied degrees of epithelial-mesenchymal transition and CD44+CD24- stemness
Source: Oncotarget. 2017 May 19;8(37):61163–80. doi: 10.18632/oncotarget.18022 (PMC5617414; doi:10.18632/oncotarget.18022)

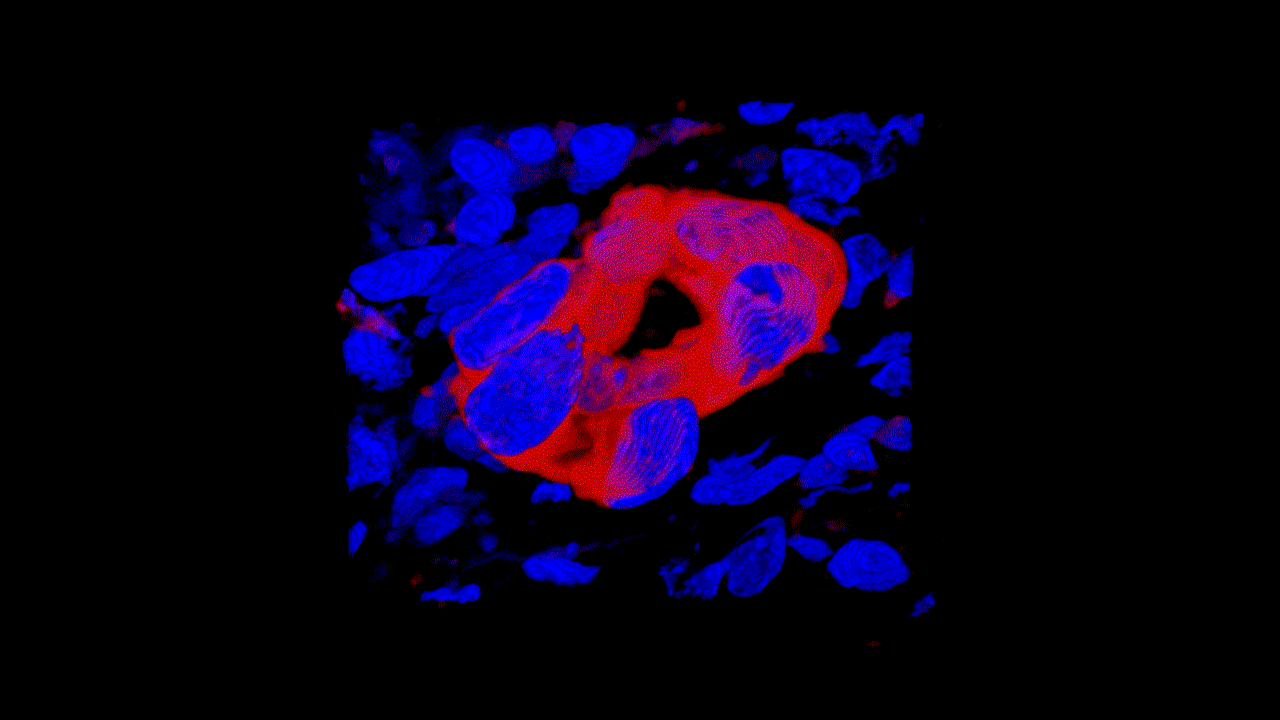

Supplement: Supplementary file 2 [file oncotarget-08-61163-s002.gif]

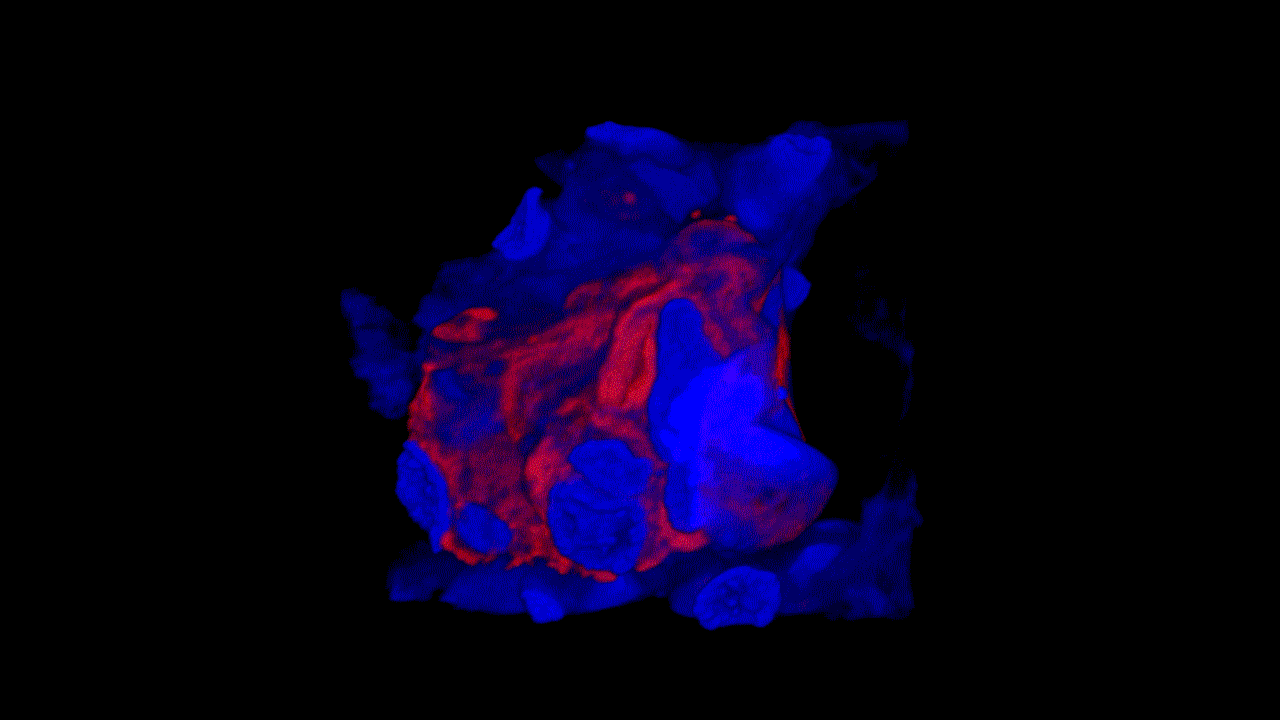

Supplement: Supplementary file 3 [file oncotarget-08-61163-s003.gif]

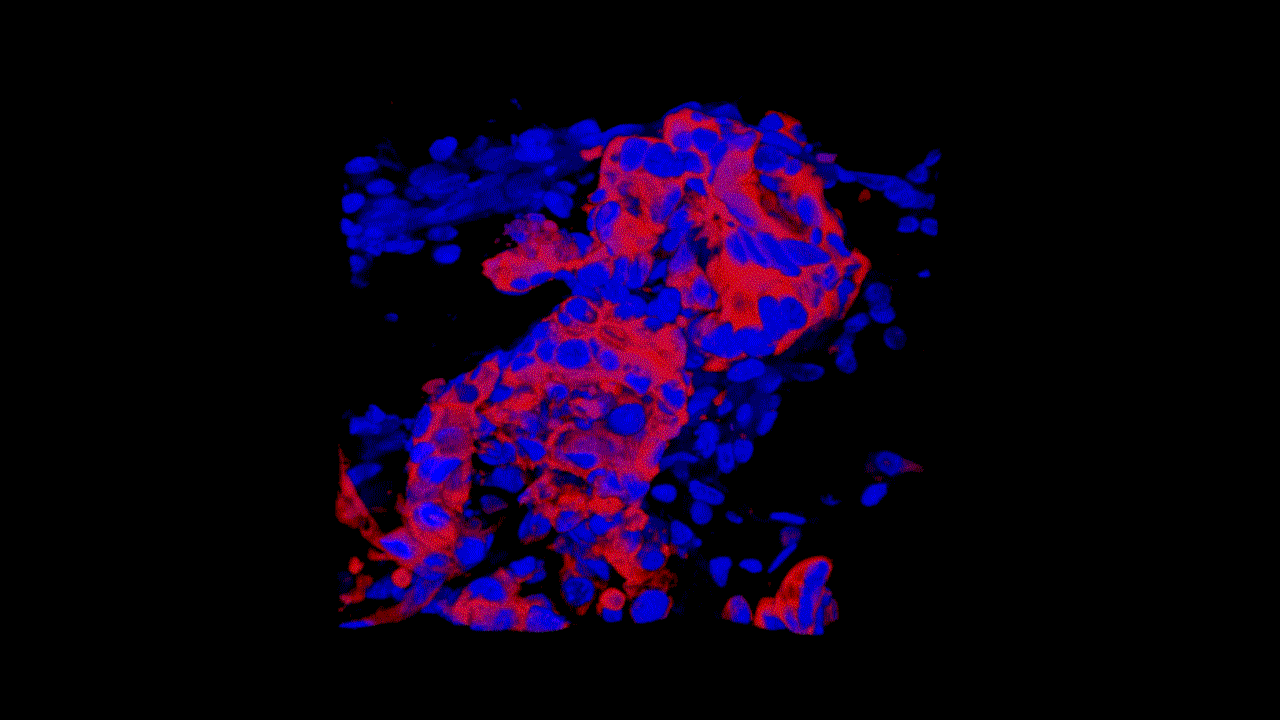

Supplement: Supplementary file 4 [file oncotarget-08-61163-s004.gif]

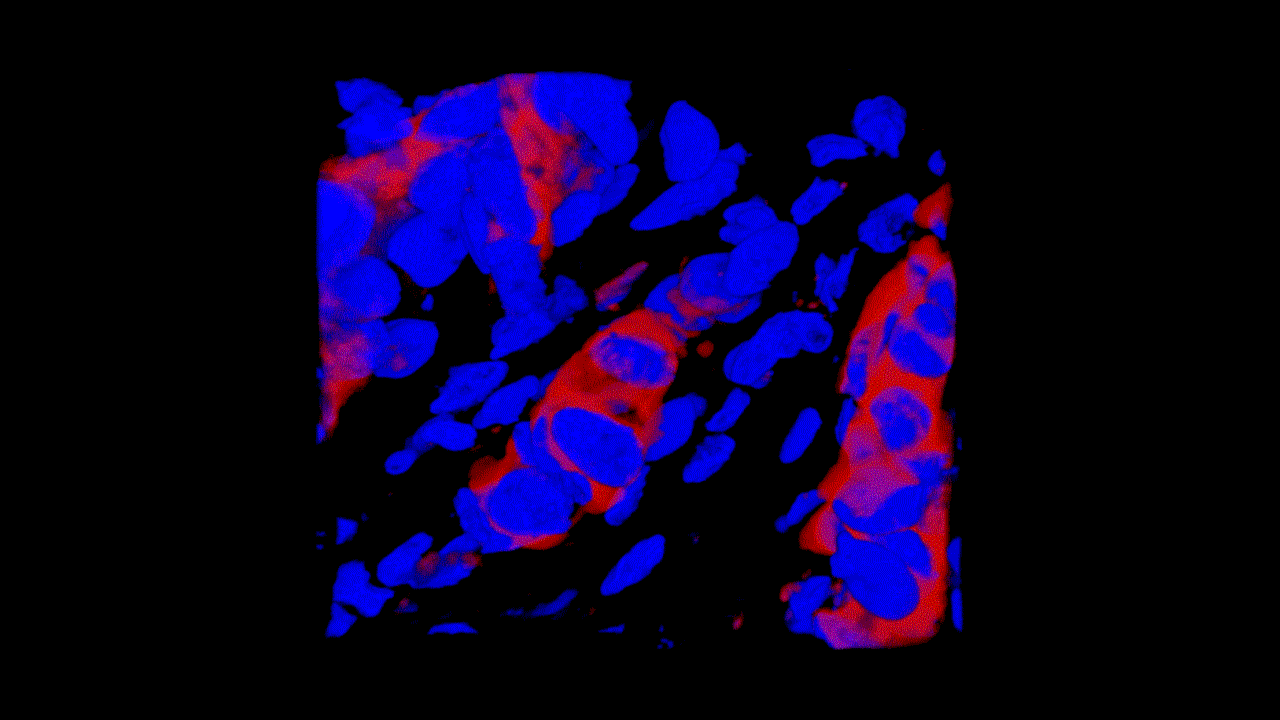

Supplement: Supplementary file 5 [file oncotarget-08-61163-s005.gif]

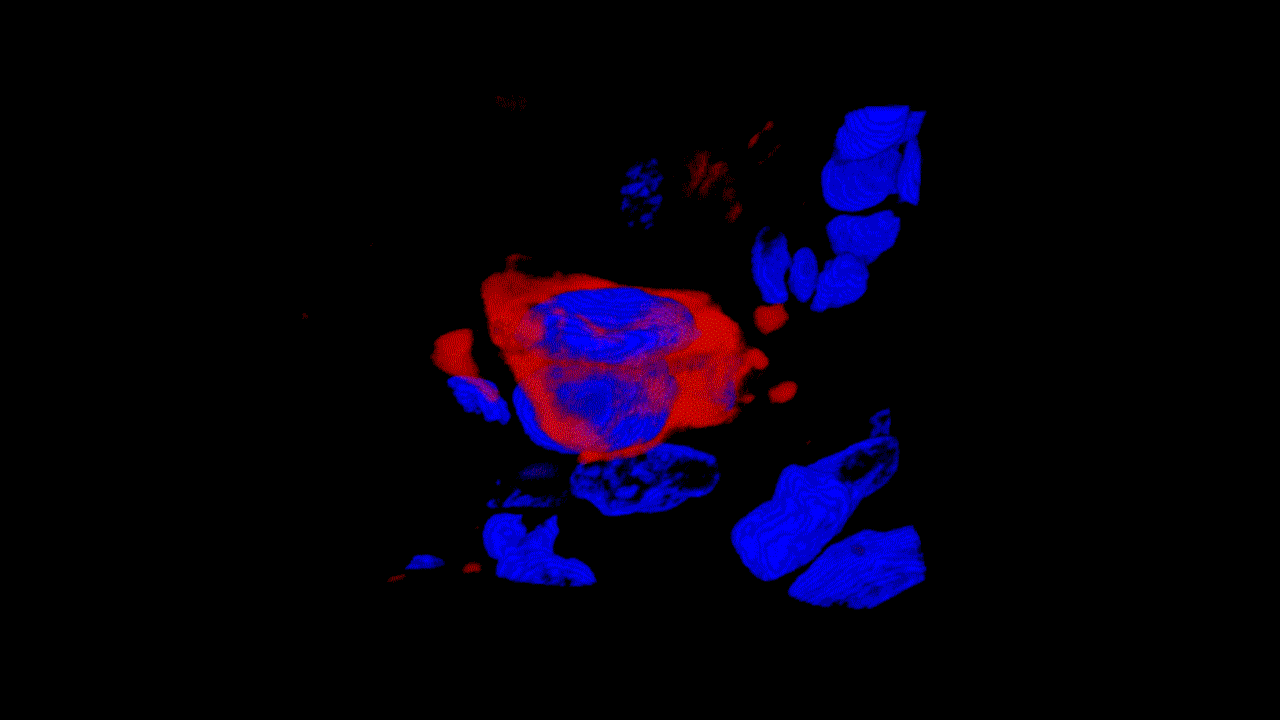

Supplement: Supplementary file 6 [file oncotarget-08-61163-s006.gif]

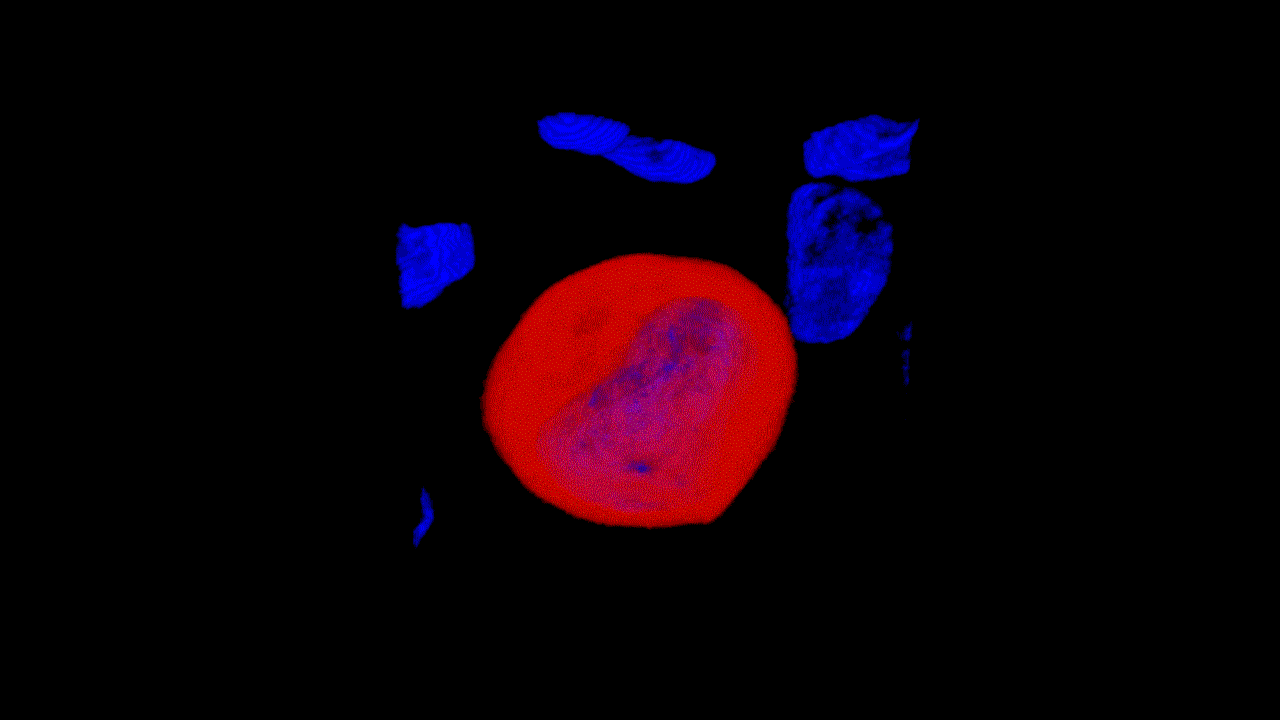

Supplement: Supplementary file 7 [file oncotarget-08-61163-s007.gif]
