# Supplementary material for: Clinically relevant morphological structures in breast cancer represent transcriptionally distinct tumor cell populations with varied degrees of epithelial-mesenchymal transition and CD44+CD24- stemness
Source: Oncotarget. 2017 May 19;8(37):61163–80. doi: 10.18632/oncotarget.18022 (PMC5617414; doi:10.18632/oncotarget.18022)
Supplement: Supplementary file 11 [file oncotarget-08-61163-s011.doc]

|  | **Gene symbol** | **Full name/ Synonyms** | **Protein function** |
| --- | --- | --- | --- |
| Epithelial | *CDH1* | Cadherin 1/ E-cadherin | A classical cadherin, component of adherens junctions |
|  | *CD24* | CD24 molecule | A mucin-like cell surface protein that plays a role in leukocyte signal transduction, regulation of B-cell apoptosis, leukocyte adhesion, maintaining cancer cell growth, anchorage-independent proliferation, and survival. |
|  | *EPCAM* | Epithelial cell adhesion molecule | A homotypic calcium-independent cell adhesion molecule that is functional antagonist for classic cadherin-mediated cell–cell interactions and plays a morphoregulatory role in normal epithelia and stem/progenitor cells and drives tumor progression. |
|  | *KRT5* | Keratin 5 | The intermediate filament component of cytoskeleton of basal epithelial cells |
|  | *LCN2* | Lipocalin 2/ Neutrophil gelatinase-associated lipocalin (NGAL) | An iron-trafficking protein involved in innate immune response |
|  | *TP63* | Tumor protein p63 | A transcription factor that plays a role in different processes from skin development and adult stem/progenitor cell regulation to apoptotic function, oocyte integrity, heart development, and premature aging. |
|  | *TNFSF10* | TNF superfamily member 10/ TNF-related apoptosis-inducing ligand (TRAIL), CD253 | A ligand that induces apoptosis primarily in transformed in tumor cells |
|  | *SLPI* | Secretory leukocyte protease inhibitor (SLPI)/ Antileukoproteinase | A secreted inhibitor which protects epithelial tissues from serine proteases |
|  | *IL1B* | Interleukin 1 Beta | A cytokine that mediates the inflammatory response and is involved in a variety of cellular activities, including cell proliferation, differentiation, and apoptosis |
|  | *S100A8* | S100 Calcium Binding Protein A8 | A calcium- and zinc-binding protein that regulates inflammatory processes and immune response |
|  | *JUP* | Junction Plakoglobin/ γ-catenin | The cytoplasmic component of desmosomes and adherens junctions structures |
|  | *TJP1* | Tight Junction Protein 1 | A protein located on a cytoplasmic membrane surface of intercellular tight junctions |
|  | *CTNNA3* | Catenin Alpha 3 | A member of the vinculin/alpha-catenin family, cell-cell adhesion |
|  | *CLDN3* | Claudin 3 | Components of tight junctions |
|  | *OCLN* | Occludin |  |

**Supplementary Table 6: The characterization of genes used for the analysis of epithelial and mesenchymal features in different morphological structures of breast tumors**

| Mesenchymal | *ABCA6* | | ATP Binding Cassette Subfamily A Member 6 | ABC transporter |
| --- | --- | --- | --- | --- |
|  | *DCN* | | Decorin | A small leucine-rich proteoglycan that plays a role in collagen fibril assembly |
|  | *IL1R1* | | Interleukin 1 Receptor Type 1 | A cytokine receptor, important mediator of many cytokine-induced immune and inflammatory responses |
|  | *PCOLCE* | | Procollagen C-Endopeptidase Enhancer | A glycoprotein which binds and drives the enzymatic cleavage of type I procollagen and heightens C-proteinase activity |
|  | *WNT5A* | | Wnt Family Member 5A | A member of the WNT family that is implicated in oncogenesis and in several developmental processes, including regulation of cell fate and patterning during embryogenesis. |
|  | *VIM* | | Vimentin | The class-III intermediate filament found in various non-epithelial cells, especially mesenchymal cells |
|  | *ZEB2* | | Zinc Finger E-Box Binding Homeobox 2 | A transcription factor that regulates early growth and development |
|  | *MMP14* | | Matrix Metallopeptidase 14/ Membrane-Type-1 Matrix Metalloproteinase (MT1-MMP) | A metalloproteinase that is involved in remodeling of extracellular matrix, actin cytoskeleton reorganization, and cell growth and migration |
|  | Trailblazer cells | *DOCK10* | Dedicator Of Cytokinesis 10 | A member of the dedicator of cytokinesis protein family |
|  |  | *ITGA11* | Integrin Subunit Alpha 11 | A receptor for collagen, adhesion of cells to the extracellular matrix |
|  |  | *DAB2* | DAB2, clathrin adaptor protein | A mitogen-responsive phosphoprotein that participates in signal transduction pathways (e.g. TGFβ-signaling) and regulates different cell processes: cell positioning, macrophage adhesion, protein trafficking, the homeostasis of epithelial differentiation etc. |
|  |  | *PDGFRA* | Platelet Derived Growth Factor Receptor Alpha | A mitogen for cells of mesenchymal origin that plays a role in organ development, wound healing, and tumor progression |
|  |  | *VASN* | Vasorin | This protein negatively modulates TGFβ-signaling and protects cells against TNFα- and hypoxia-induced apoptosis |
|  |  | *PLPP3* | Phospholipid Phosphatase 3/ Phosphatidic Acid Phosphatase Type 2B (PPAP2B) | An enzyme that hydrolyzes extracellular lysophosphatidic acid (LPA) and short-chain phosphatidic acid and regulate vascular and embryonic development by inhibiting LPA signaling |
|  |  | *LPAR1* | Lysophosphatidic Acid Receptor 1 | A member of a group of lysophospholipid receptors that mediate diverse biologic functions, including proliferation, platelet aggregation, smooth muscle contraction, inhibition of neuroblastoma cell differentiation, chemotaxis, and tumor cell invasion. |

The information presented is given in accordance with HUGO Gene Nomenclature Committee ([http://www.genenames.org](http://www.genenames.org/)), GeneCards: The Human Gene Database (<http://www.genecards.org/>), OMIM – Online Mendelian Inheritance in Man ([http://omim.org](http://omim.org/)), The Gene Wiki (<https://en.wikipedia.org/wiki/Portal:Gene_Wiki>) and papers by Prunier and Howe, J Biol Chem 2005, Smith et al., Cancer Res 2006, Krautzberger et al., Gene Expr Patterns 2012, and Schnell et al., Biochim Biophys Acta 2013.
