# Supplementary material for: Clinically relevant morphological structures in breast cancer represent transcriptionally distinct tumor cell populations with varied degrees of epithelial-mesenchymal transition and CD44+CD24- stemness
Source: Oncotarget. 2017 May 19;8(37):61163–80. doi: 10.18632/oncotarget.18022 (PMC5617414; doi:10.18632/oncotarget.18022)
Supplement: Supplementary file 12 [file oncotarget-08-61163-s012.doc]

**Supplementary Table 7:** Clinicopathological characteristics of breast cancer patients

| **Cases** | | **Age at diagnosis (yr)** | | **Grade** | | **TNM** | | **Focality** | | **Molecular subtype** | **ER** | | **PR** | | **HER2** | | **Ki-67 (%)** | | | **Morphological structures** | |
| --- | --- | --- | --- | --- | --- | --- | --- | --- | --- | --- | --- | --- | --- | --- | --- | --- | --- | --- | --- | --- | --- |
| 1 | | 46 | | 2 | | T2N0M0 | | UF | | Lum B HER2− | + | | + | | 0 | | 34 | | | all structures | |
| 2 | | 40 | | 2 | | T2N2M0 | | UF | | Lum B HER2− | + | | + | | 1+ | | 24 | | | all structures | |
| 3 | | 49 | | 2 | | T2N0M0 | | UF | | Lum B HER2+ | + | | + | | 2+ (FISH+) | | 45 | | | all structures | |
| 4 | | 59 | | 2 | | T2N3M0 | | UF | | Lum B HER2+ | + | | + | | 2+ (FISH+) | | 38 | | | all structures | |
| 5 | | 49 | | 2 | | T1N0M0 | | UF | | Lum B HER2− | + | | + | | 1+ | | 37 | | | all structures | |
| 6 | | 65 | | 2 | | Т1N0M0 | | UF | | Lum A | + | | + | | 1+ | | 19 | | | all structures | |
| 7 | | 42 | | 3 | | T1N1M0 | | UF | | Lum B HER2− | + | | + | | 1+ | | 24 | | | alv, sol, trab, discr | |
| 8 | | 53 | | 1 | | T1N0M0 | | UF | | Lum B HER2− | + | | − | | 0 | | 34 | | | alv, sol, trab, discr | |
| 9 | | 62 | | 2 | | T2N0M0 | | UF | | Lum B HER2− | + | | − | | 0 | | 27 | | | all structures | |
| 10 | | 65 | | 1 | | T2N0M0 | | UF | | Lum B HER2− | + | | + | | 1+ | | 25 | | | alv, sol, trab, discr | |
| 11 | | 63 | | 2 | | T2N0M0 | | UF | | Lum B HER2+ | + | | + | | 2+ (FISH+) | | 29 | | | all structures | |
| 12 | | 61 | | 2 | | T1bN1M0 | | UF | | Lum A | + | | − | | 1+ | | 15 | | | all structures | |
| 13 | | 59 | | 2 | | T1bN0M0 | | UF | | Lum B HER2− | + | | + | | 1+ | | 69 | | | alv, sol, trab, discr | |
| 14 | | 42 | | 1 | | Т1bN4M1 | | MF | | Lum A | + | | + | | 1+ | | 8 | | | all structures | |
| 15 | | 39 | | 2 | | T2N1M0 | | UF | | Lum B HER2− | + | | + | | 1+ | | 33 | | | all structures | |
| 16 | | 53 | | 2 | | T2N0M0 | | UF | | Lum B HER2+ | + | | + | | 2+ (FISH+) | | 11 | | | all structures | |
| 17 | | 56 | | 2 | | T2N1M0 | | UF | | Lum B HER2+ | + | | − | | 3+ | | 60 | | | all structures | |
| 18 | | 68 | | 3 | | T1bN0M0 | | UF | | Lum B HER2− | + | | − | | 2+ (FISH−) | | 28 | | | all structures | |
| 19 | | 68 | | 2 | | T1bN0M0 | | UF | | Lum B HER2− | + | | + | | 1+ | | 33 | | | all structures | |
| 20 | | 58 | | 2 | | T1bN1M0 | | UF | | Lum B HER2+ | + | | + | | 3+ | | 59 | | | sol, discr | |
| 21 | | 53 | | 2 | | T1bN1M1 | | MF | | Lum B HER2+ | + | | + | | 2+ (FISH+) | | 16.6 | | | sol, discr | |
| 22 | | 68 | | 2 | | Т2N0M0 | | UF | | Lum A | − | | + | | 1+ | | 5 | | | alv, sol, trab, discr | |
| 23 | | 61 | | 2 | | Т2N1M0 | | UF | | Lum A | + | | − | | 0 | | 14 | | | all structures | |
| 24 | | 45 | | 2 | | Т2N0M0 | | UF | | Lum B HER2− | + | | + | | 1+ | | 24 | | | all structures | |
| 25 | | 41 | | 2 | | T2N0M0 | | MF | | Lum A | + | | + | | 1+ | | 16 | | | all structures | |
| 26 | | 68 | | 1 | | T1N0M0 | | UF | | Lum A | + | | + | | 0 | | 18 | | | all structures | |
| 27 | | 50 | | 2 | | T2N0M0 | | UF | | Lum B HER2− | + | | + | | 0 | | 26 | | | all structures | |
| 28 | 56 | | 2 | | T2N0M0 | | UF | | Lum B HER2− | | | + | | + | | 1+ | | 44 | alv, sol, trab, discr | |  |

| 29 | 48 | 2 | T1N0M0 | MF | Lum A | + | + | 1+ | 17 | all structures |
| --- | --- | --- | --- | --- | --- | --- | --- | --- | --- | --- |
| 30 | 53 | 2 | T2N1M0 | UF | Lum A | + | + | 1+ | 15 | all structures |
| 31 | 36 | 2 | T2N0M0 | UF | Lum A | + | + | 1+ | 18 | all structures |
| 32 | 42 | 2 | T1aN0M1 | UF | Lum B HER2− | + | + | 1+ | 26 | alv, sol, trab, discr |
| 33 | 67 | 1 | T1NхМ0 | UF | Lum A | + | + | 1+ | 15 | all structures |
| 34 | 66 | 2 | T2N0M0 | UF | Lum B HER2− | + | + | 1+ | 38 | sol, discr |
| 35 | 60 | 3 | T2N3M0 | UF | Lum B HER2− | + | + | 0 | 65 | alv, sol, trab, discr |
| 36 | 44 | 2 | T2N0M0 | UF | Lum B HER2− | + | + | 1+ | 55 | sol |
| 37 | 61 | 2 | T1N1M0 | UF | Lum B HER2− | + | + | 1+ | 33 | all structures |
| 38 | 45 | 2 | T3N1M0 | UF | Lum B HER2+ | + | + | 2+ (FISH+) | 40 | all structures |
| 39 | 40 | 2 | Т1N0М0 | UF | Lum B HER2− | + | + | 1+ | 30 | all structures |
| 40 | 68 | 2 | Т2N1М0 | UF | Lum B | + | + | 1+ | 17 | all structures |

UF: unifocal; MF: multifocal; Lum: luminal; yr: years; ER: estrogen receptors; PR: progesterone receptors; TNM: tumor-node-metastasis classification; "+": presence; "−": absence; FISH: fluorescence *in situ* hybridization; tub: tubular; alv: alveolar; sol: solid; trab: trabecular; discr: discrete.
